# Chronic Ca^2+^ imaging of cortical neurons with long-term expression of GCaMP-X

**DOI:** 10.7554/eLife.76691

**Published:** 2022-10-05

**Authors:** Jinli Geng, Yingjun Tang, Zhen Yu, Yunming Gao, Wenxiang Li, Yitong Lu, Bo Wang, Huiming Zhou, Ping Li, Nan Liu, Ping Wang, Yubo Fan, Yaxiong Yang, Zengcai V Guo, Xiaodong Liu

**Affiliations:** 1 Advanced Innovation Center for Biomedical Engineering, School of Biological Science and Medical Engineering, School of Engineering Medicine, Key Laboratory for Biomechanics and Mechanobiology of Ministry of Education, Beihang University Beijing China; 2 X-Laboratory for Ion-Channel Engineering, Beihang University Beijing China; 3 Tsinghua-Peking Joint Center for Life Sciences, IDG/McGovern Institute for Brain Research, School of Medicine, Tsinghua University Beijing China; 4 Center for Life Sciences, School of Life Sciences, Yunnan University Kunming China; 5 Key Laboratory for Biomedical Engineering of Ministry of Education, Zhejiang University Hangzhou China; Columbia University United States; The University of Texas at Austin United States

**Keywords:** genetically encoded calcium indicators, cortical neurons, calmodulin, calcium oscillations, in vivo calcium imaging, neurite outgrowth, Mouse

## Abstract

Dynamic Ca^2+^ signals reflect acute changes in membrane excitability, and also mediate signaling cascades in chronic processes. In both cases, chronic Ca^2+^ imaging is often desired, but challenged by the cytotoxicity intrinsic to calmodulin (CaM)-based GCaMP, a series of genetically-encoded Ca^2+^ indicators that have been widely applied. Here, we demonstrate the performance of GCaMP-X in chronic Ca^2+^ imaging of cortical neurons, where GCaMP-X by design is to eliminate the unwanted interactions between the conventional GCaMP and endogenous (apo)CaM-binding proteins. By expressing in adult mice at high levels over an extended time frame, GCaMP-X showed less damage and improved performance in two-photon imaging of sensory (whisker-deflection) responses or spontaneous Ca^2+^ fluctuations, in comparison with GCaMP. Chronic Ca^2+^ imaging of one month or longer was conducted for cultured cortical neurons expressing GCaMP-X, unveiling that spontaneous/local Ca^2+^ transients progressively developed into autonomous/global Ca^2+^ oscillations. Along with the morphological indices of neurite length and soma size, the major metrics of oscillatory Ca^2+^, including rate, amplitude and synchrony were also examined. Dysregulations of both neuritogenesis and Ca^2+^ oscillations became discernible around 2–3 weeks after virus injection or drug induction to express GCaMP in newborn or mature neurons, which were exacerbated by stronger or prolonged expression of GCaMP. In contrast, neurons expressing GCaMP-X were significantly less damaged or perturbed, altogether highlighting the unique importance of oscillatory Ca^2+^ to neural development and neuronal health. In summary, GCaMP-X provides a viable solution for Ca^2+^ imaging applications involving long-time and/or high-level expression of Ca^2+^ probes.

## Introduction

Ca^2+^ signals play pivotal roles in the brain, closely involved in membrane excitability, sensory transduction, synaptic transmission, neural development, and plasticity (Berridge et al., 2003). Ca^2+^ dysregulations are linked with the mental disorders including Parkinson’s diseases, Alzheimer’s diseases, epilepsy, and schizophrenia (Chan et al., 2007; Fernández de Sevilla et al., 2006; Khan et al., 2020; Liebscher et al., 2016), suggesting that multiple factors and cascades may converge to Ca^2+^ as one of the central factors underlying brain diseases, referred as the calcium hypothesis (Berridge, 2010). According to their downstream consequences, cellular Ca^2+^ signals could be categorized as genomic versus non-genomic, to reflect the fact that in some cases where gene expressions are regulated (chronic) versus in other cases only the (acute) functions of existing proteins are concerned.

Acutely, cellular Ca^2+^ reflects single-neuron activities, such as spontaneous fluctuations and stimulus-evoked responses (Chen et al., 2013; O’Banion and Yasuda, 2020). Ca^2+^ imaging is often utilized to measure neuronal excitability, as one alternative to electrical recording. In fact, genetically encoded Ca^2+^ indicators (GECIs) represented by GCaMP, based on CaM (calmodulin) and Ca^2+^/CaM-binding motif M13, have been broadly applied to monitor neurons and other excitable cells (Akerboom et al., 2012; Chen et al., 2013; Dana et al., 2019; Nakai et al., 2001; Tallini et al., 2006; Tian et al., 2009; Yang et al., 2018). In addition to a faithful index of acute responses (in the timescale of seconds/minutes, such as a burst of action potentials), Ca^2+^ is often tightly coupled to various chronic effects or processes, for example, Ca^2+^-dependent gene transcription and expression, neurite outgrowth or pruning, long-term potentiation or depression, learning and memory, and neural degeneration (O’Banion and Yasuda, 2020). Therefore, it is highly desirable to monitor the long-term Ca^2+^ dynamics (days/weeks or longer) for cells, tissues, organs, or even whole organisms, which would greatly facilitate mechanistic understanding of the genomic/chronic roles of Ca^2+^ in diverse pathophysiology (Garcia et al., 2017). Meanwhile, back to the context of non-genomic Ca^2+^, longitudinal imaging may have a broad scope of applications where long-term changes in responses or behaviors are of interest. In parallel with Ca^2+^ imaging, different types of electrodes, such as MEA (multiple electrode array) and flexible electronics, have been extensively deployed for cultured neurons, brain slices, live animals, or human brains to record neural activities in the format of neuronal action potentials, local-field potentials, and EEG (Hong and Lieber, 2019). The goal is to monitor neural activities across multiple days, weeks, or even the entire lifespan in the studies of training/behaviors, retina/vision, brain disorders, addictions, and pharmacological and interventional therapeutics (Aramuni and Griesbeck, 2013; Couto et al., 2021). GECIs hold great promise to avoid chronic immune responses and recoding instability that electrodes are often encountered with (Aramuni and Griesbeck, 2013). Indeed, GCaMP and other GECIs have been demonstrated as more advantageous methods over electrodes or dyes during chronic imaging of neurons (Aoki et al., 2017; Murphy et al., 2020; Tian et al., 2009). Unfortunately, neural toxicities often accompany long-term expression of GCaMP or chronic GCaMP imaging with either virus infected (Chen et al., 2013; Tian et al., 2009; Yang et al., 2018) or transgenic neurons (Steinmetz et al., 2017).

Perturbing L-type Ca_V_1 channels and presumably other (apo)CaM-binding proteins, GCaMP indicators cause side-effects in neurons which have been documented especially for enhanced or prolonged expressions. The unwanted molecular events of GCaMP may or may not be manifested as the toxic effects at the cellular or system levels. For instance, when employing viral infection, imaging experiments are restricted within the empirical time window and dosage range to alleviate nuclear-filling of GCaMP (Resendez et al., 2016); or for transgenic mice, special promoters, conditional expression, and other tactics are utilized to reduce the expression level/time of GCaMP (Madisen et al., 2015). With such work-around solutions, rich information and rapid progress in neurosciences have been achieved by GCaMP imaging. At a cost, special cautions and procedures are required due to GCaMP toxicity, for example virus dilution trials (Resendez et al., 2016), which often cause inconvenience in experiments besides other potential problems (e.g., nucleus-filling or low SNR).

Under the testing conditions in this work, neuronal perturbations were evidenced from GCaMP: with either earlier or newer versions, for either viral or transgenic expression, either in vitro (cultured neurons) or in vivo (living mice), and on either acute sensory response to whisker deflection or spontaneous oscillation encoding genomic Ca^2+^. GCaMP-X has been designed to resolve these perturbations by eliminating unwanted interferences with endogenous (apo)calmodulin signaling. For in vivo imaging beyond the safe time window (3-week extension) and dosage (10-fold higher), GCaMP-X outperformed GCaMP in recapitulating both sensory responses to whisker stimulation and autonomous Ca^2+^ fluctuations. In cultured cortical neurons, GCaMP of strong and/or prolonged expression caused the damage to neurites accompanied by aberrant Ca^2+^ oscillations, all overcome by GCaMP-X as a simple solution, which also highlights the importance of oscillatory Ca^2+^ to neurons both in vitro and in vivo.

## Results

### Design principles of GCaMP-X validated by newer GCaMP versions

**Figure 1.**
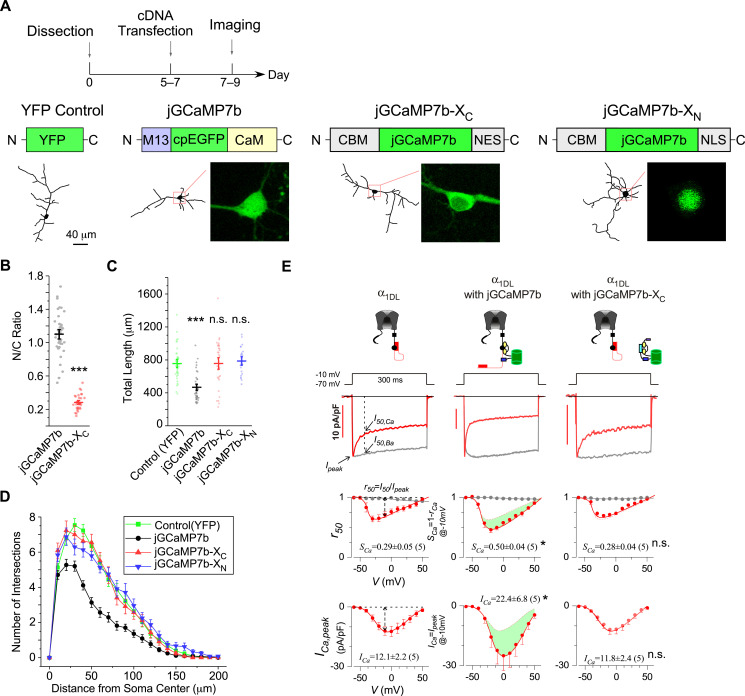
The design principles applicable to jGCaMP7 and jGCaMP7-X. (**A**) Cultured cortical neurons from newborn mice were transiently transfected with YFP, jGCaMP7b, jGCaMP7b-X_C_, or jGCaMP7b-X_N_, respectively, on DIV 5–7, then imaged by confocal microscopy on DIV 7–9. As illustrated, apoCaM-binding motif (CBM) was fused onto N-terminus of GCaMP and the tags of localization signals (nuclear export signal or nuclear localization signal, NES/NLS) were fused to the C-terminus of GCaMP to construct GCaMP-X_C_ or GCaMP-X_N_, respectively. Neurite tracing and subcellular GCaMP distributions are shown below. (**B**) N/C ratio of jGCaMP7b or jGCaMP7b-X_C_ in neurons, by calculating the nucleus versus cytosol ratio of fluorescence intensities. Total length (**C**) and *Sholl* analysis (**D**) for cortical neurons expressing YFP or GCaMP variants. Two independent experiments from two independent culture preparations (**A–D**). (**E**) Electrophysiological validations of jGCaMP7b versus jGCaMP7b-X_C_ with recombinant Ca_V_1.3 channels. Full-length Ca_V_1.3 channels (α_1DL_) were expressed in HEK293 cells alone (left) or with jCaMP7b (middle) or with jGCaMP7b-X_C_ (right). At −10 mV, Ca^2+^ current traces (red, with scale bars indicating current amplitudes) and Ba^2+^ current traces (gray, rescaled) are shown. *S*_Ca_ and *I*_Ca_ (quantified by the equations shown in the first column) are the indices of calcium-dependent inactivation and voltage-dependent activation, respectively. Cell numbers are indicated in the parentheses right after the values. Standard error of the mean (SEM) and two-tailed unpaired Student’s *t*-test (**B**) or one-way analysis of variance (ANOVA) followed by Bonferroni for post hoc tests (**C, E**) (criteria of significance: *p < 0.05; ***p < 0.001; *n.s.* denotes ‘not significant’) were applied.

Recently, GCaMP has been updated to its newest versions jGCaMP7 (but also see the bioRxiv preprint of jGCaMP8 [Zhang et al., 2021]), with enhanced sensing performance in multiple aspects over the previous GCaMP6 (Dana et al., 2019; Grødem et al., 2021). Considering that the design of jGCaMP7 is also on the basis of CaM, we postulated that jGCaMP7-contained apoCaM would have similar problems to those of earlier GCaMPs. We chose jGCaMP7b to further validate the design principles of neuron-compatible GCaMP-X established in our previous work (Yang et al., 2018). Following the protocol of transient transfection (Figure 1A), jGCaMP7b accumulated into the nuclei in a substantial subpopulation of cortical neurons indexed by N/C (nucleus/cytosol) ratio (Figure 1B), considered as the hallmark of GCaMP side-effects. Notably, jGCaMP7b exhibited even more severe nuclear accumulation than other GCaMP variants, which may account for the nuclear jGCaMP7b evidenced in vivo (Dana et al., 2019). Accordingly, the total length (Figure 1C) and the complexity (Figure 1D) of neurites were significantly reduced in jGCaMP7b-expressing neurons. The apoCaM-binding motif (CBM) and the localization tags were then appended onto jGCaMP7b, following the design of GCaMP-X (Yang et al., 2018), to construct jGCaMP7b-X_C_ and jGCaMP7b-X_N_ for cytosolic and nuclear Ca^2+^ imaging, respectively. GCaMP-X is supposed to eliminate its binding to apoCaM targets in neurons and reduce the cytotoxicity intrinsic to GCaMP, a critical issue to long-term Ca^2+^ monitoring. Depicted by neurite tracing (Figure 1A), both cytosolic and nuclear versions of jGCaMP7-X have greatly enhanced the compatibility with neurons. In fact, neurons expressing jGCaMP7-X were essentially indistinguishable from GFP control neurons, in direct contrast to the neurons transfected with jGCaMP7 of the same amount of cDNA (Figure 1C, D). In light of electrophysiological analyses on calmodulation of Ca_V_1 channels (Ben-Johny and Yue, 2014; Yang et al., 2018), we examined the effects of jGCaMP7b on recombinant Ca_V_1.3 channels in HEK293 cells. jGCaMP7b significantly altered the major properties of Ca_V_1.3 gating, that is, both inactivation and activation were enhanced (Figure 1E). The expression level is a critical factor to evaluate the side-effects of GCaMP. We then examined the actual levels of expressed proteins in HEK293 cells when transiently transfected with the same amount of cDNA for different indicators (Figure 1—figure supplement 1). Demonstrated by both western blotting and immunohistochemistry, jGCaMP7b in HEK293 cells was expressed either in the cytosol or in the nucleus at the same levels as jGCaMP7b-X_C_ or jGCaMP7b-X_N_, respectively. Likewise, jGCaMP7 and jGCaMP7-X were at the same cytosolic or nuclear levels of expression when transiently transfected into neurons (Figure 1—figure supplement 2). Moreover, by coexpressing jGCaMP7b-X_C_ and jGCaMP7b-X_N_, the total expression level estimated by immunostaining was even higher than that of jGCaMP7b, the latter of which caused significant damage to neurites whereas the total neurite length of jGCaMP7b-X neurons was about the same as GFP control neurons. Therefore, the expression levels of GCaMP-X versus GCaMP did not underlie their differential performances.

**Figure 1—figure supplement 1.**
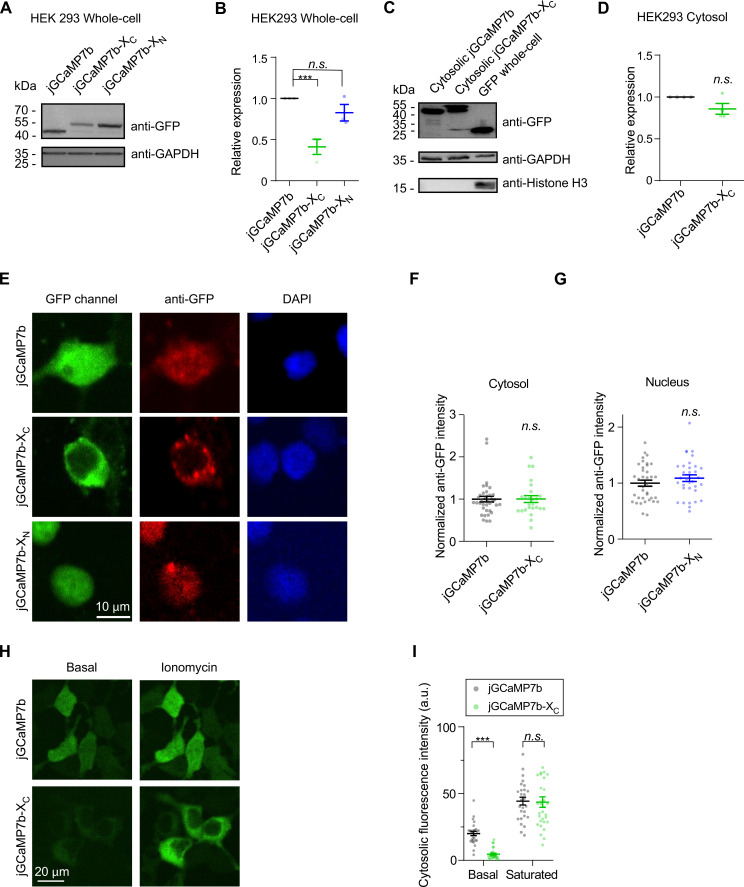
Expression levels of indicators in HEK293 cells. (**A–D**) jGCaMP7b, jGCaMP7b-X_C_, and jGCaMP7b-X_N_ were transiently transfected into HEK293 cells to examine the level of protein expression by western blot. Whole-cell extracts (**A, B**) and cytosolic extracts (**C, D**) were separately examined. The anti-GFP bands and anti-GAPDH bands indicate the expression levels and the amount of inputs, respectively. The anti-Histone H3 bands served as the nuclear marker to confirm the efficacy of the fractionation. The relative expression levels were normalized to the jGCaMP7b control for the whole cell (**B**) or cytosol (**D**) proteins. (**E–G**) Comparison of protein expression of indicators in HEK293 cells by immunostaining. HEK cells expressing jGCaMP7b, jGCaMP7b-X_C_, and jGCaMP7b-X_N_ were stained with anti-GFP (red channel) and DAPI (4′,6-diamidino-2-phenylindole, blue channel) to measure the level of expression and illustrate the nuclear envelop, respectively. Confocal images in GFP channels indicate GCaMP fluorescence (**E**). The anti-GFP signals were normalized over jGCaMP7b to compare the expression levels. The cytosolic and nuclear levels of jGCaMP7b were compared with jGCaMP7b-X_C_ (**F**) and jGCaMP7b-X_N_ (**G**), respectively. (**H, I**) HEK293 cells expressing jGCaMP7b or jGCaMP7b-X_C_ were stimulated by 2.5 μM ionomycin (a potent calcium ionophore) to saturate the indicators (**H**). Indexed by cytosolic fluorescence intensity, the two indicators were compared at the basal or saturated state (**I**), suggesting that it is possible to use probe fluorescence to estimate the expression level if the conditions are carefully controlled. Three independent experiments from three independent samples. Standard error of the mean (SEM) and one-way analysis of variance (ANOVA) followed by Bonferroni for post hoc tests (**B**) or two-tailed unpaired Student’s *t*-test (**D, F, G, I**) (criteria of significance: ***p < 0.001; *n.s*. represents ‘not significant’) were applied. Figure 1—figure supplement 1—source data 1.Source data for Figure 1—figure supplement 1A.Original uncropped western blotting gels with indication of the cropped areas. Figure 1—figure supplement 1—source data 2.Source data for Figure 1—figure supplement 1C.Original uncropped western blotting gels with indication of the cropped areas.

**Figure 1—figure supplement 2.**
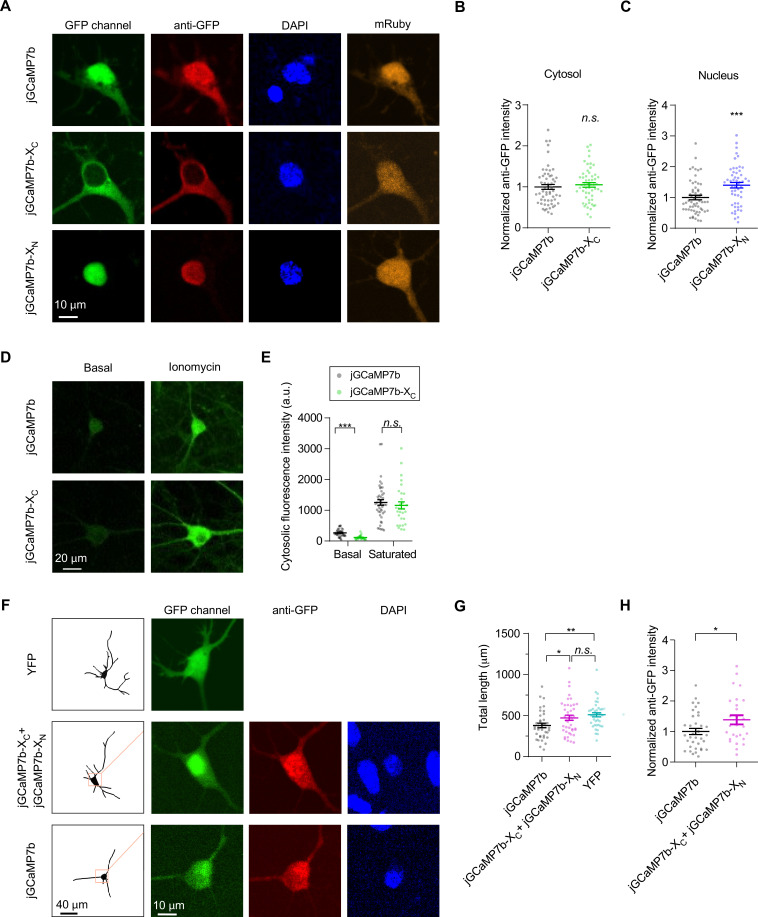
Expression levels of indicators in cultured cortical neurons. (**A–C**) The indicators of jGCaMP7b, jGCaMP7b-X_C_, and jGCaMP7b-X_N_ expressed in cultured cortical neurons were examined by immunostaining. Similar to HEK293 cells, cultured cortical neurons from newborn mice transiently transfected by jGCaMP7b, jGCaMP7b-X_C_, or jGCaMP7b-X_N_ were stained by anti-GFP (red channel) and DAPI (blue channel) to measure the protein expression and illustrate the nuclear envelop, respectively. Confocal images in GFP channels pointed out the positive neurons. The anti-GFP signals were normalized over the jGCaMP7b groups. The cytosolic and nuclear levels of jGCaMP7b were compared with jGCaMP7b-X_C_ (**B**) and jGCaMP7b-X_N_ (**C**), respectively. (**D, E**) Cortical neurons expressing jGCaMP7b or jGCaMP7b-X_C_ were stimulated by 2.5 μM ionomycin (**D**). Indexed by cytosolic fluorescence intensity, the two indicators were compared at the basal or saturated state (**E**). (**F–H**) jGCaMP7b-X_C_ and jGCaMP7b-X_N_ were cotransfected into cortical neurons to compare with jGCaMP7b. Cultured cortical neurons expressing YFP served as the control. Anti-GFP and DAPI fluorescence images indicate the protein expression and the nuclear envelop, respectively (**F**). The total length of neurites was calculated and compared for the three groups of neurons (**G**). Indexed by normalized anti-GFP intensity (over jGCaMP7b) cells coexpressing jGCaMP7b-X_C_ and jGCaMP7b-X_N_ resulted in an even higher level of probe expression than jGCaMP7b. Three independent experiments from three independent culture preparations. Standard error of the mean (SEM) and two-tailed unpaired Student’s *t*-test (**B, C, E, H**) or one-way analysis of variance (ANOVA) followed by Bonferroni for post hoc tests (**G**) (criteria of significance: *p < 0.05, **p < 0.01, ***p < 0.001; *n.s*. represents ‘not significant’) were applied.

**Figure 1—figure supplement 3.**
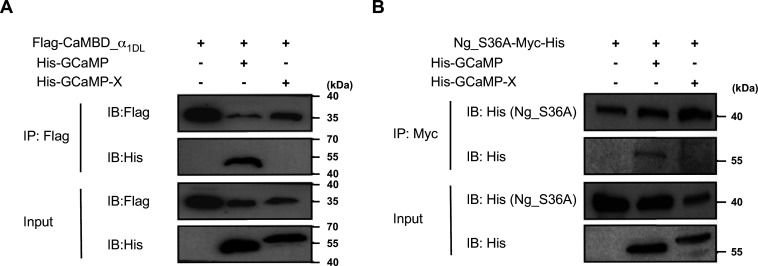
Differential interactions of GCaMP versus GCaMP-X with apoCaM-binding proteins. Coimmunoprecipitation results of His-tagged GCaMP or GCaMP-X_C_ with Flag-tagged apoCaM-binding domain of α_1DL_ (CaMBD_α_1DL_) (**A**), or with Myc- and His-tagged YFP-Ng_S36A (**B**). HEK293 cell lysates were added with 5 mM EGTA; *n* = 3 replicates. Figure 1—figure supplement 3—source data 1.Source data for Figure 1—figure supplement 3A, B.Original uncropped western blotting gels with indication of the cropped areas.

Notably, by coimmunoprecipitation GCaMP bound Ca_V_1.3 (α_1D_) at its apoCaM-binding domain whereas GCaMP-X was found no binding (Figure 1—figure supplement 3). Similarly, neurogranin, an important postsynaptic apoCaM-binding protein (Gerendasy and Sutcliffe, 1997), was unveiled to bind GCaMP but not GCaMP-X. Thus, the above direct evidence of molecular interactions further consolidated the design principles of GCaMP-X, serving as the major candidate mechanism of cellular GCaMP toxicities.

### Acute sensory responses monitored by viral expression of GCaMP-X in vivo

**Figure 2.**
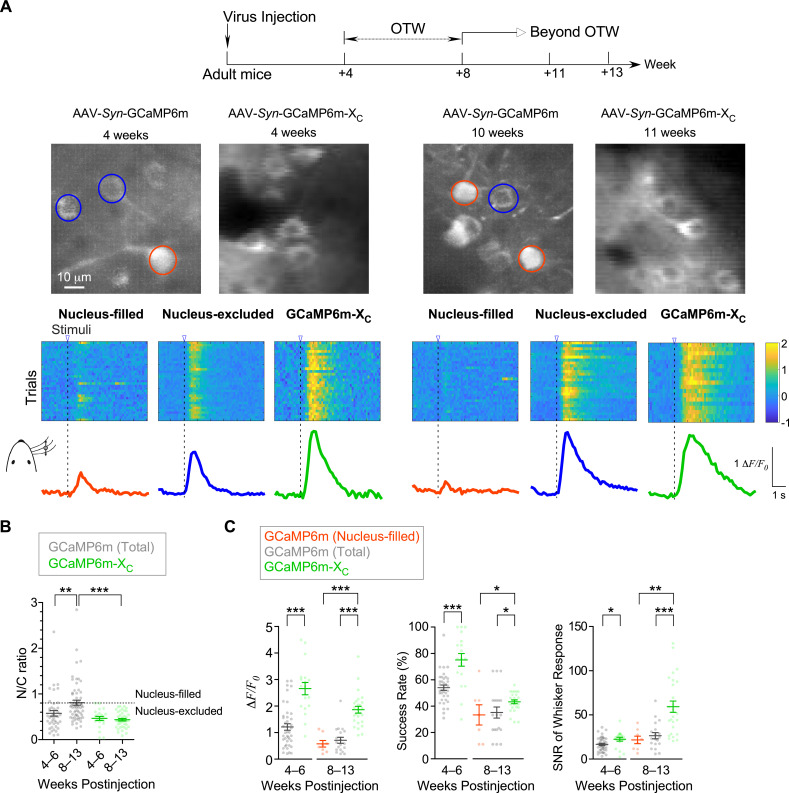
In vivo Ca^2+^ imaging of sensory-evoked responses in cortical neurons virally infected with GCaMP-X versus GCaMP. (**A**) Ca^2+^ dynamics of GCaMP6m and GCaMP6m-X_C_ in S1 primary somatosensory cortex under whisker stimulation by in vivo two-photon Ca^2+^ imaging. 4–6 and 8–13 weeks postinjection were considered as optimal time window (OTW) or beyond OTW, respectively. The orange and blue circles in the representative two-photon images indicate nucleus-filled and nucleus-excluded GCaMP6m, respectively. The colored scale bar indicates the fluorescence intensity of Ca^2+^ probes. Multiple trials of the same neuron are shown for nucleus-filled GCaMP6m, nucleus-excluded GCaMP6m, or GCaMP6m-X_C_. The averaged Ca^2+^ responses are shown at the bottom. (**B**) Subcellular distributions of GCaMP6m and GCaMP6m-X_C_ in vivo within OTW or beyond OTW. Neurons were divided into two distinct subgroups, nucleus-excluded and nucleus-filled, by applying the cutoff value (N/C ratio) of 0.8. (**C**) Responses to repetitive whisker stimuli were evaluated and compared for GCaMP6m nucleus-filled neurons, total GCaMP6m neurons, and GCaMP6m-X_C_. The average amplitude (*ΔF*/*F*_*0*_) (left), success rate of the trials (middle) and SNR (right) during whisker stimuli were compared. Data were obtained from five or four mice for GCaMP6m and GCaMP6m-X_C_, respectively. Standard error of the mean (SEM) and one-way analysis of variance (ANOVA) followed by Bonferroni for post hoc tests (criteria of significance: *p < 0.05; **p < 0.01; ***p < 0.001) were calculated when applicable.

GECIs including GCaMP have been widely applied to monitor neuronal responses to various stimuli. Due to the cytotoxicity known from the very early versions of GCaMP, in vivo imaging experiments are normally required to conduct within the time window. In practice, an optimal time window (OTW) is about 3–8 weeks postinjection for GCaMP-infected neurons of live mice (Chen et al., 2013; Huber et al., 2012; Resendez et al., 2016), in order to achieve substantial levels of GCaMP expression and fluorescence but not too high levels prone to side-effects. Here, we investigated into the Ca^2+^ dynamics under whisker stimulation within or beyond OTW by applying the adeno-associated viruses (AAVs) of GCaMP6m or GCaMP6m-X_C_ with the neurospecific *Syn* promoter to S1 primary somatosensory cortex in the brain of adult mice (2-month age or older) (Figure 2A). To exclude the potential bias due to level of expression, GCaMP6m-X_C_ viruses (1.0 × 10^13^ v.g./ml) of higher dose than GCaMP6m (1.0 × 10^12^ v.g./ml) were injected (60 nl/injection). Progressive nuclear accumulation of GCaMP was previously reported in vivo and in vitro (Chen et al., 2013; Yang et al., 2018; Zariwala et al., 2012). Consistently, by the criteria of N/C ratio (0.8), nucleus-filling GCaMP was observed in a fraction of neurons 4–6 weeks postinjection, and the average N/C ratio was substantially increased when examined 8–13 weeks postinjection (Figure 2B). In direct contrast, no neuron expressing GCaMP6m-X_C_ fell into the nucleus-filled category even weeks beyond OTW (up to 13 weeks postinjection). In the earlier study (Chen et al., 2013), the impairment on visual responses was reported from nucleus-filled neurons after long-term expression of GCaMP6 (several months postinjection); and during the initial phase (1- to 2-month postinjection) nuclear GCaMP6 did not perturb the proper physiology of neurons. Here, nucleus-filled neurons expressing GCaMP6m (N/C ratio >0.8) started to show less responsiveness than neurons expressing GCaMP6m-X_C_ as early as within OTW (4–6 weeks) (Figure 2A), which may underlie the lower amplitude of Ca^2+^ responses (*ΔF*/*F*_*0*_) averaged over the whole population of neurons (Figure 2C). Another two indices of success rate and SNR (signal to noise ratio) were also consistent with the above notion that within OTW neurons filled with nuclear GCaMP might have been impaired. Beyond OTW (8–13 weeks), GCaMP6m was more frequently and clearly found in the nucleus, and the neurons exhibited more significant differences from GCaMP6m-X_C_ according to all the three indices of *ΔF*/*F*_*0*_, success rate, and SNR.

Our data have extended the advantages of GCaMP-X over GCaMP from in vitro onto in vivo. Collectively, neurons may suffer from GCaMP side-effects either within or beyond OTW, for which nuclear GCaMP expression appears to be a critical factor. GCaMP-X outperformed GCaMP in imaging sensory-evoked Ca^2+^ dynamics especially beyond OTW, which provides a simple but promising solution for long-term Ca^2+^ imaging.

### Long-term monitoring of Ca^2+^ oscillations by GCaMP-X_C_ in vitro

**Figure 3.**
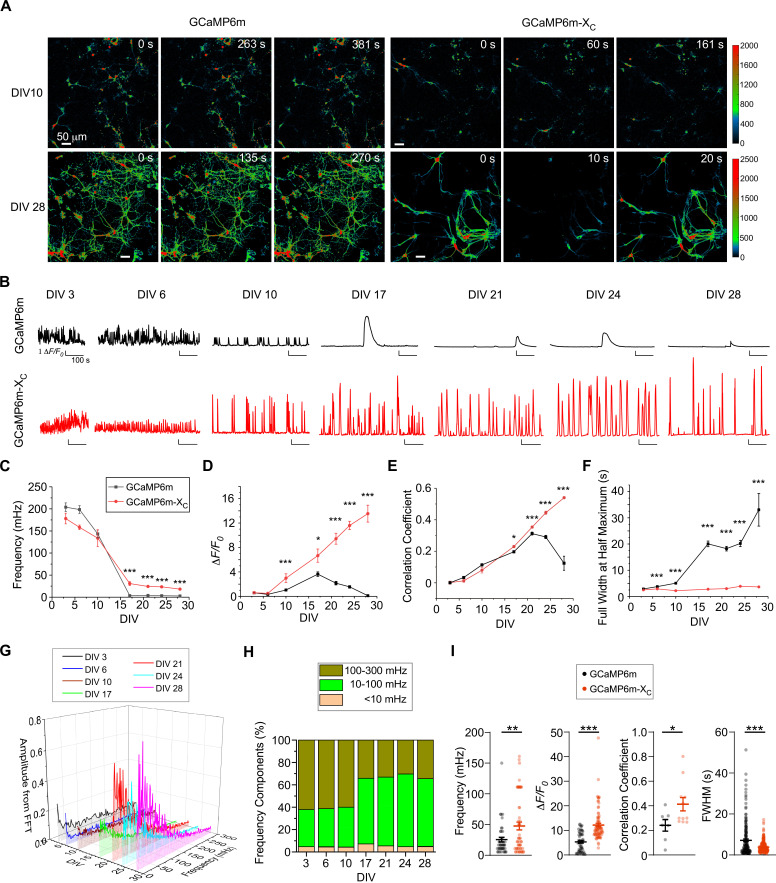
Chronic Ca^2+^ fluorescence imaging for autonomous Ca^2+^ oscillations in cultured cortical neurons. (**A**) Time-lapse images of cultured cortical neurons infected with AAV-*Syn*-GCaMP6m or AAV-*Syn*-GCaMP6m-X_C_. Spontaneous Ca^2+^ activities by the two probes are shown for DIV 10 and DIV 28. See Figure 3—videos 1–3 for details. Ca^2+^ signals (color coded) were monitored by the confocal microscope with a live-cell imaging chamber to maintain the cell culture conditions (37°C, 5% CO_2_, 97–100% humidity) at different timepoints. (**B**) Representative traces of Ca^2+^ activities in cultured cortical neurons expressing GCaMP6m (upper) or GCaMP6m-X_C_ (lower) from DIV 3 to DIV 28. Temporal profiles of key indices measured from spontaneous Ca^2+^, including the average frequency (10^–3^ Hz or mHz) (**C**) and peak amplitude (*ΔF*/*F*_*0*_, **D**); synchrony (quantified by the mean of correlation coefficient per view, **E**) and full width at half maximum (FWHM, **F**). (**G**) Power spectral analyses by FFT (Fast Fourier Transformation) for Ca^2+^ traces of cortical neurons from DIV 3 to DIV 28. (**H**) Frequency components in percentage. By integrating the absolute amplitudes over each frequency band (**G**), three major bands are shown:<10 mHz (ultra-slow), 10–100 mHz (slow), and 100–300 mHz (fast), where the 10–100 mHz band indicates the major component. (**I**) Summary over three independent experiments with three independent culture preparations (see Figure 3—figure supplement 2 for the other two experiments). Key indices of frequency (DIV 35), *ΔF*/*F*_*0*_ (DIV 28), correlation coefficient per view (DIV 28) and FWHM (DIV 28) were calculated and compared for GCaMP6m versus GCaMP6m-X_C_. Standard error of the mean (SEM) and the Student’s *t*-test (two-tailed unpaired with criteria of significance: *p < 0.05; **p < 0.01; ***p < 0.001) were calculated when applicable.

Before proceeding further with in vivo GCaMP-X imaging, we decided to conduct in-depth examinations on the long-term performance of GCaMP-X under in vitro conditions. Of note, slow Ca^2+^ oscillations have been observed from a variety of excitable or non-excitable cells (Uhlén and Fritz, 2010). In neurons, oscillatory Ca^2+^ signals can increase the efficiency and specificity of gene expression (Dolmetsch et al., 1998; Li et al., 1998), thus playing important role in neuronal functions, development, morphology, and even general health (Kamijo et al., 2018; Nicotera and Orrenius, 1998; Toth et al., 2016). Spontaneous electrical activities are initiated at the early stage of neural development, and subsequently become more synchronized (Luhmann et al., 2016; Spitzer, 2006). Such longitudinal processes of oscillatory Ca^2+^ and neuritogenesis which are mechanistically coupled may serves as a perfect scenario to demonstrate the side-effects of GCaMP on neurons. We then hypothesized that GCaMP-X versus GCaMP neurons would make a clear difference in their chronic recordings of oscillatory Ca^2+^ signals. Meanwhile, GCaMP-X with enhanced neuron compatibility is expected to provide new insights into the roles of spontaneous Ca^2+^ oscillations in neuronal morphology (Gomez and Zheng, 2006; Rosenberg and Spitzer, 2011). To monitor such Ca^2+^ dynamics in vitro, the AAVs carrying GCaMP6m or GCaMP6m-X_C_ (for in vitro use) were equally (1 μl, 1 × 10^12^ v.g./ml) added to the cortical neurons of neonatal mice (DIV 0, 0-day in vitro) which were maintained and examined till DIV 28 (Figure 3A, B). Fluctuations of Ca^2+^ activities were perceivable starting from the first week (DIV 3 and DIV 6) with GCaMP6m-X_C_, in the pattern of high-frequency, low-amplitude, and unsynchronized signals. On DIV 10, the oscillation frequency decreased while the amplitude was increased with an enhanced level of synchronization. On DIV 28, Ca^2+^ oscillations of individual or subgrouped neurons became widely synchronized featuring robust spikes and slow frequency (Figure 3—video 1), indicative of the formation of neural circuitry. In contrast, Ca^2+^ signals were severely distorted in GCaMP6m-infected DIV-10 neurons. Despite that the performance of GCaMP6m in the first week resembled GCaMP6m-X_C_, longer expression time of GCaMP6m resulted in altered patterns of Ca^2+^ oscillations (Figure 3—videos 2 and 3). One major abnormity was the substantial reduction in oscillatory activities of GCaMP6m-expressing neurons, which was manifested after DIV 10 by much longer intervals between two adjacent peaks and much smaller amplitudes in average. Occasionally, abnormal Ca^2+^ spikes with ultralong lasting duration could be observed on DIV 17 (Figure 3B, Figure 3—video 2). Around DIV 28, cease of Ca^2+^ oscillations and broken neurites were often evidenced (Figure 3—video 3). We then further analyzed oscillatory Ca^2+^ signals by the frequency and other key indices across the timespan from DIV 3 up to DIV 28. Statistical results demonstrated that the frequency of Ca^2+^ fluctuation with GCaMP6m-X_C_ was about 150 mHz during the first week, then gradually declined to the plateau around 20 mHz (Figure 3C). Meanwhile, the peak amplitude exhibited a rising trend in the neurons expressing GCaMP6m-X_C_ across the full term (Figure 3D). In contrast, both the frequency and the amplitude of Ca^2+^ oscillations acquired by GCaMP6m were drastically changed after DIV 17, and then even more deteriorated later in that the oscillation was less and less recognizable and eventually halted on DIV 28 (Figure 3C, D). Synchronization is one major hallmark of autonomous Ca^2+^ oscillations, which was evaluated by the mean of correlation coefficient. As demonstrated by the temporal profiles of correlation coefficients, the comparison between GCaMP6m versus GCaMP6m-X_C_ unveiled a crucial phase turning from increasing to decreasing synchronization around DIV 17–21 in GCaMP6m-expressing neurons (Figure 3E). Likewise, the full width at half maximum (FWHM), another index of oscillatory waveforms, was aberrantly wider for GCaMP6m than GCaMP6m-X_C_, becoming noticeable on DIV 10, and much more pronounced (10-fold) later on (Figure 3F). Collectively from these indices, GCaMP indeed caused progressive damage on cortical neurons along with the culturing time or developmental stages; in contrast, GCaMP6m-X_C_ has overcome nearly all the above negative effects, emerging as a promising tool for chronic Ca^2+^ imaging with enhanced neural compatibility. Also, Fast Fourier Transformation (FFT) was applied to the Ca^2+^ waveforms acquired by GCaMP6m-X_C_ (Figure 3G and Figure 3—figure supplement 1). The distribution of frequency components started to change during DIV 10–17, when slow Ca^2+^ oscillations of 10–100 mHz appeared to be the dominant form (Figure 3H). Based on separate preparations of neurons, we conducted another two experiments to compare GCaMP6m and GCaMP6m-X_C_ up to DIV 42 (Figure 3—figure supplement 2). Statistical results from these data on frequency, *ΔF*/*F*_*0*_, correlation coefficient per view and FWHM support that GCaMP-X outperforms GCaMP in chronic Ca^2+^ imaging of cultured neurons (Figure 3I). The newer probes of jGCaMP7b and jGCaMP7b-X_C_ resulted in differential performance that jGCaMP7b-X_C_ was much less toxic, consistent with the above notion (Figure 3—figure supplement 3).

**Figure 3—figure supplement 1.**
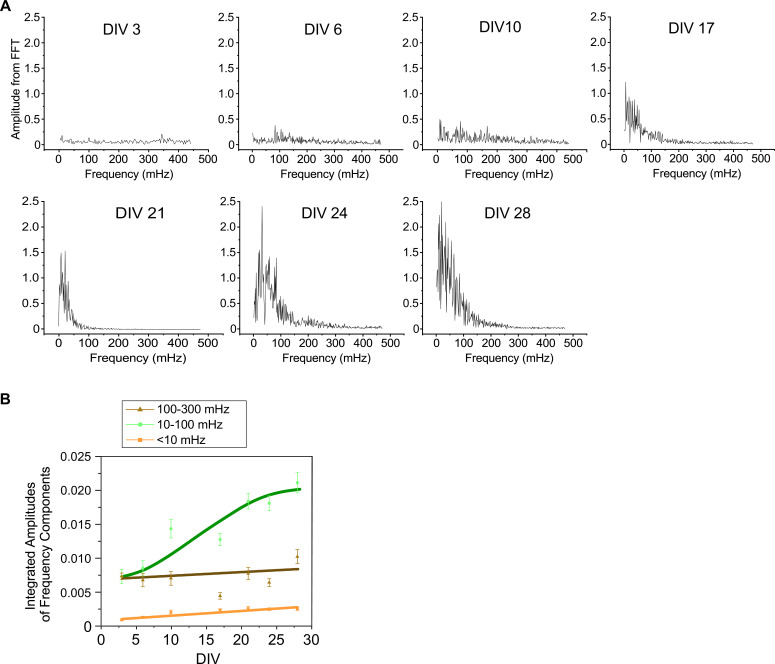
Spectral analysis for Ca^2+^ waveforms acquired by GCaMP-X. (**A**) Representative FFT-based power spectra of individual neurons at different timepoints (DIV). Neurons from DIV 3 to DIV 10 exhibited low FFT amplitudes, whereas neurons from DIV 21 to DIV 28 showed significantly large amplitudes with the central frequency around 10–100 mHz. DIV 17 appeared to be the transition time for neurons to develop from weak oscillations (DIV 3 to DIV 10) to strong oscillations (DIV 21 to DIV 28). (**B**) Temporal profiles for the major frequency components of Ca^2+^ oscillations. Three frequency components including 100–300 mHz (fast), 10–100 mHz (slow), and <10 mHz (ultraslow) were summarized (*n* = 3 individual experiments). Frequency components were evaluated by the amplitude integration over each frequency band in FFT spectra. Standard error of the mean (SEM) was calculated when applicable.

**Figure 3—figure supplement 2.**
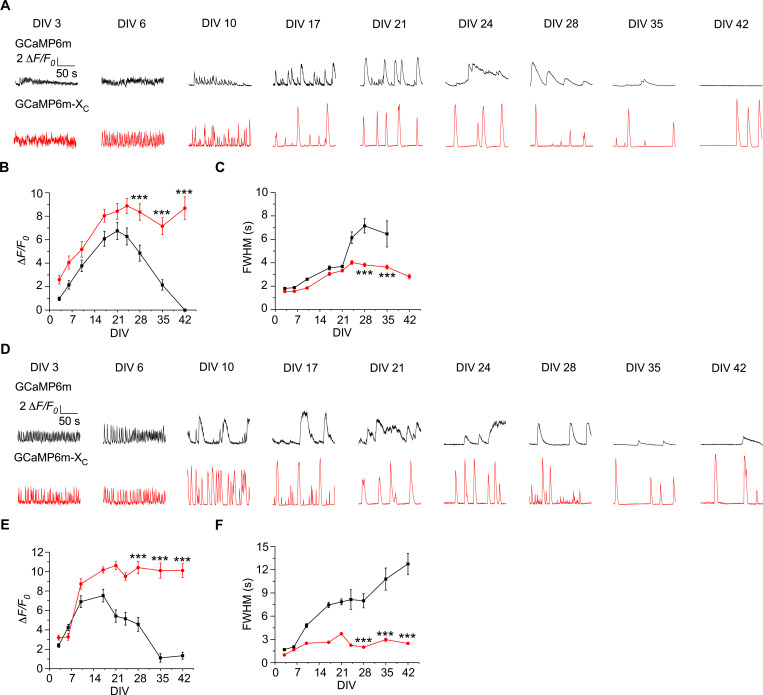
Ca^2+^ oscillations in long-term cultured cortical neurons expressing GCaMP6m or GCaMP6m-X_C_ in vitro. (**A–C**) Ca^2+^ activities in cultured cortical neurons expressing GCaMP6m (black) or GCaMP6m-X_C_ (red), respectively. Exemplar traces from DIV 3 to DIV 42 (**A**), summarized by peak amplitudes (*ΔF*/*F*_*0*_, **B**) and full width at half maximum (FWHM, **C**). Note that GCaMP6m on DIV 42 did not record any noticeable response. (**D–F**) Another set of Ca^2+^ oscillation data and analyses over multiple weeks similar to (**A–C**). Standard error of the mean (SEM) and Student’s *t*-test (two-tailed unpaired with criteria of significance: ***p < 0.001) were calculated when applicable.

**Figure 3—figure supplement 3.**
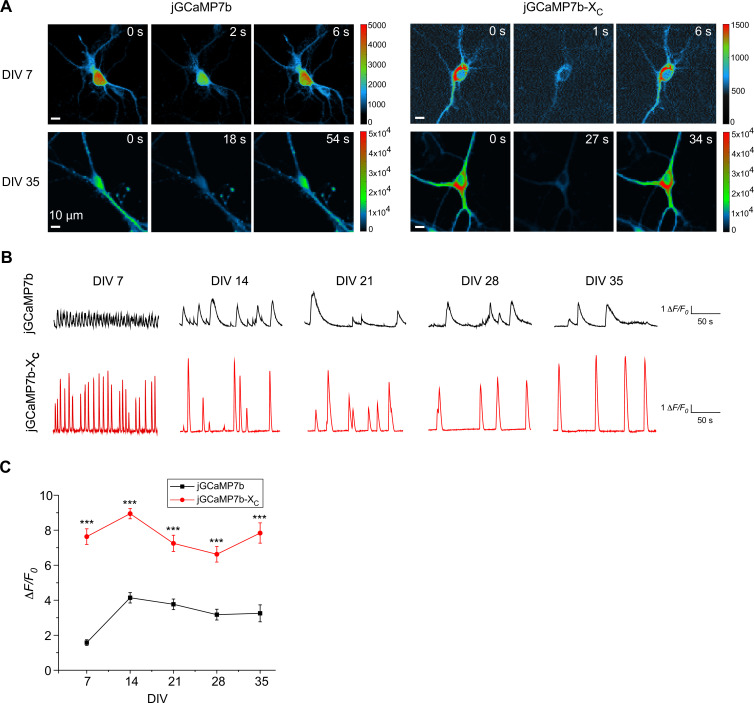
Ca^2+^ oscillations in cultured cortical neurons expressing jGCaMP7b or jGCaMP7b-X_C_. (**A**) Representative Ca^2+^ fluorescence images (color coded) of cultured cortical neurons expressing jGCaMP7b or jGCaMP7b-X_C_ on DIV 7 or DIV 35. (**B**) Representative Ca^2+^ activity traces of cultured cortical neurons expressing jGCaMP7b (black) or jGCaMP7b-X_C_ (red) across 5 weeks (from DIV 7 to DIV 35). (**C**) Summarized peak amplitudes in time-dependent profiles (*ΔF*/*F*_*0*_). Three independent experiments from three independent culture preparations. Standard error of the mean (SEM) and Student’s *t*-test (two-tailed unpaired with criteria of significance: ***p < 0.001) were calculated when applicable.

**Figure 3—figure supplement 4.**
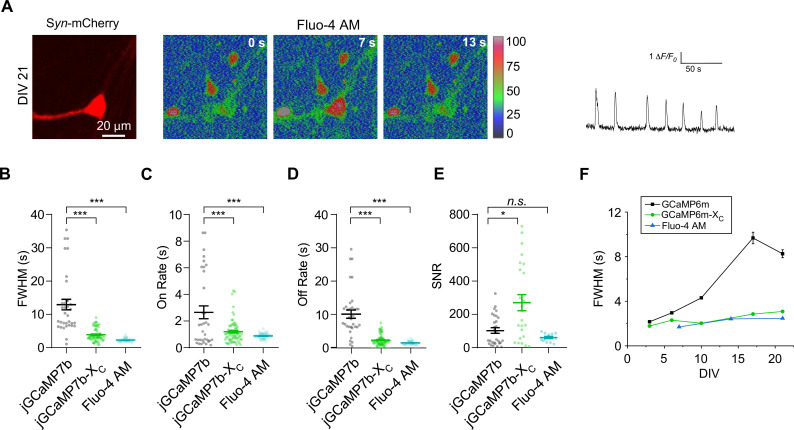
Ca^2+^ oscillations in cultured cortical neurons imaged by Fluo-4 AM. (**A**) Ca^2+^ signals of cultured cortical neurons on DIV 21. Neurons were infected with pLenti-*Syn*-mCherry virus to illustrate the cell body (left). Ca^2+^ fluorescence intensities from neurons loaded with Fluo-4 AM were color coded (middle). A trace of Fluo-4 AM fluorescence represents the Ca^2+^ signals in a neuron (right). Summary of the key indices: FWHM (in seconds, **B**), on rate (in seconds, **C**), off rate (in seconds, **D**), and SNR (**E**) of Ca^2+^ signals acquired by jGCaMP7b, jGCaMP7b-X_C_, or Fluo-4 AM. (**F**) Temporal profiles of FWHM measured from spontaneous Ca^2+^ oscillations acquired by GCaMP6m, GCaMP6m-X_C_, or Fluo-4 AM. Standard error of the mean (SEM) and one-way analysis of variance (ANOVA) followed by Bonferroni for post hoc tests (criteria of significance: *p < 0.05, ***p < 0.001; *n.s*. represents ‘not significant’) were applied.

**Figure 3—video 1.** Spontaneous Ca^2+^ oscillation of cultured cortical neurons virally expressing GCaMP6m-X_C_ on DIV 28. Neurons exhibited highly synchronized Ca^2+^ oscillations with robust spikes and complicated neurite connections. Video plays at 60× speed, with each frame in the size of 512×512 pixels or 631×631 µm^2^.

**Figure 3—video 2.** Spontaneous Ca^2+^ oscillation of cultured cortical neurons virally expressing GCaMP6m on DIV 17. Neurons exhibited ultralong lasting Ca^2+^ signals which may be associated with apoptotic death of neurons. Video plays at 60× speed, with each frame in the size of 512×512 pixels or 631×631 µm^2^.

**Figure 3—video 3.** Spontaneous Ca^2+^ oscillation of cultured cortical neurons virally expressing GCaMP6m on DIV 28. Most of the neurons were broken and no longer exhibited Ca^2+^ oscillation. Video plays at 60× speed, with each frame in the size of 512×512 pixels or 631×631 µm^2^.

In summary, the same set of neurons were chronically monitored with GCaMP-X across the development stages from newborn to mature, by which the temporal profiles of the major characteristics were achieved for oscillatory Ca^2+^ signals in cultured cortical neurons. As one additional control, Fluo-4 AM (Ca^2+^ dye) imaging was conducted for synchronized Ca^2+^ oscillations on DIV 21 (Figure 3—figure supplement 4). The key parameters of Ca^2+^ dynamics measured by jGCaMP7b-X_C_ were much closer to those by Fluo-4 AM than jGCaMP7b, supporting that jGCaMP7b-X_C_ is less toxic than jGCaMP7b. Minor to moderate differences still existed between Fluo-4 AM and jGCaMP7b-X_C_, which were more likely attributed to intrinsic probe properties (e.g., Ca^2+^-sensing kinetics) rather than neuronal toxicities.

### Close correlations between autonomous Ca^2+^ oscillations and neuronal morphology in vitro

Spontaneous Ca^2+^ oscillations, the slow periodic Ca^2+^ waveforms in particular, are tightly coupled with neuronal morphology, development and neuritogenesis (Kamijo et al., 2018; Toth et al., 2016). GCaMP-X promises unprecedented opportunities for concurrent imaging of both neuronal functionalities and morphogenesis across different stages of development. Such chronic Ca^2+^ imaging is difficult to implement if using other approaches, for example, conventional GCaMP or Ca^2+^ dyes, both would cause side-effects to neurons (Smith et al., 2018; Yang et al., 2018). By taking advantage of GCaMP-X, we here aimed at the relationship between cellular Ca^2+^ and neuronal morphology.

**Figure 4.**
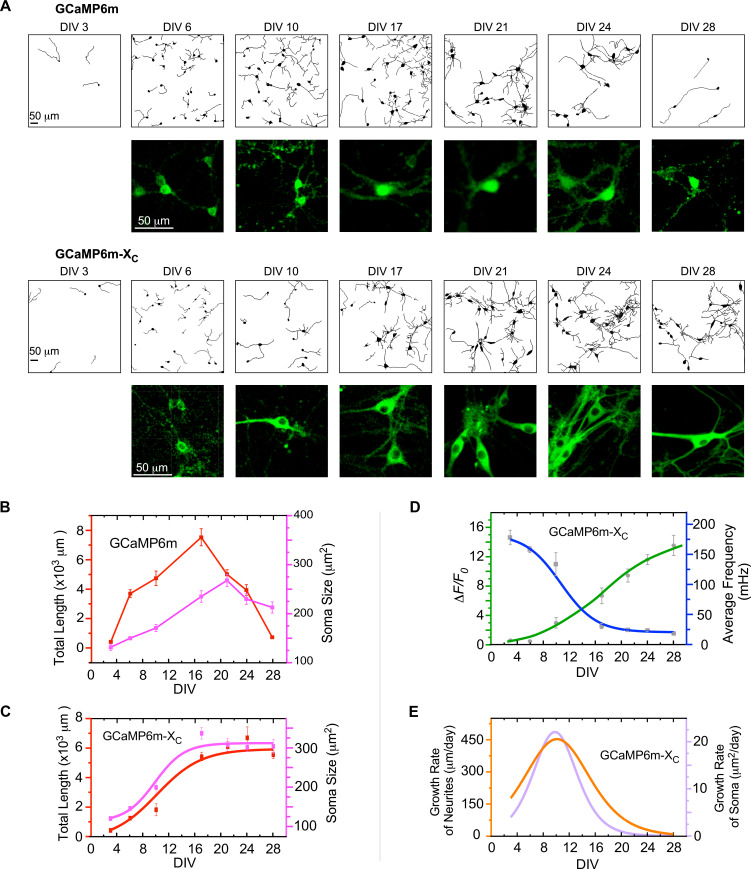
The correlations between neuronal development and Ca^2+^ oscillations unveiled by GCaMP-X. (**A**) Time-lapse images with neurite tracing for cultured cortical neurons expressing GCaMP6m (upper two rows) or GCaMP6m-X_C_ (lower two rows). Enlarged fluorescence images are to show subcellular distributions of the probes, indicative of the nuclear accumulation of GCaMP6m versus GCaMP6m-X_C_. (**B**) Temporal profiles of total neurite length per view (red) or soma size per neuron (pink) for neurons expressing GCaMP6m. (**C**) Temporal profiles of total neurite length per view (red) or soma size per neuron (pink) for neurons expressing GCaMP6m-X_C_. (**D**) The temporal profiles of the average frequency of Ca^2+^ oscillations (blue) and the peak amplitude (*ΔF*/*F*_*0*_, green), adopted from Figure 3C, D. (**E**) Temporal profiles of the growth rates (μm/day) of neurite length (orange) or soma size (purple), respectively. Analyses were performed in parallel on both Ca^2+^ waveforms and neuronal morphology based on the data obtained from the same three independent culture preparations as in Figure 3 (see Figure 4—figure supplement 1 for details on morphology data). Standard error of the mean (SEM) was calculated when applicable.

Indistinguishable from control neurons infected with GFP viruses (Figure 4—figure supplement 1), neurons expressing GCaMP6m-X_C_ followed the typical development process of neonatal neurons, including neurite elongation/arborization and soma enlargement (Figure 4A). In contrast, these developmental processes were severely impaired by virally delivered GCaMP6m, especially after DIV 14 onwards, when nuclear accumulation and neurite shortening became evident. Depicted by DIV-28 neurons with neurite tracings, GCaMP6m caused significant damage on neurite outgrowth, and to the extreme, discernable death of neurons, in contrast to GCaMP6m-X_C_ which had no apparent perturbation. In addition, the temporal profiles across the full time-course were achieved for both GCaMP6m (Figure 4B) and GCaMP6m-X_C_ (Figure 4C) by the major indices of neurite length and soma size. At the early phase (before DIV 17), no significant difference between the two groups of GCaMP6m versus GCaMP6m-X_C_ could be detected. However, toward the late stage (DIV 28 or later) of GCaMP-expressing neurons, the soma size was as small as ~200 μm^2^ in contrast to the neurons expressing GFP or GCaMP6m-X_C_ (~300 μm^2^), as confirmed by the statistical summary over three independent experiments (Figure 4—figure supplement 1). Likewise, the total neurite length of GCaMP-expressing neurons rapidly declined, whereas GCaMP-X-expressing neurons went through an initial phase (~2 weeks) of rapid outgrowth before entering into the plateau phase, forming a monotonic increasing curve. Similar to neuritogenesis, the temporal profile of soma size also exhibited an upward-plateau trend (Figure 4C). Combining the data and analyses from both developmental and functional perspectives (Figures 3 and 4), we speculated on the potential correlations between neuronal growth and spontaneous Ca^2+^ activities (Figure 4—figure supplement 2). Functionally, Ca^2+^ dynamics appeared to be either ascending (amplitude) or descending (frequency) along with the developmental stages (DIV) (Figure 4D). Roughly, the oscillation amplitude linearly (*R*^2^ = 0.84) correlated with the neurite length in total (Figure 4—figure supplement 2A). In direct contrast, the oscillation frequency and the total neurite length were inversely correlated (*R*^2^ = 0.99) (Figure 4—figure supplement 2B). Resembling the amplitude, the level of synchrony (correlation coefficient) indicative of circuitry formation was positively correlated with the total neurite length (*R*^2^ = 0.86) (Figure 4—figure supplement 2C). All these tight correlations support the notion that spontaneous Ca^2+^ activities including its mature form of synchronized Ca^2+^ oscillations may underpin neuritogenesis (Estrada et al., 2006; Gomez and Zheng, 2006; Kamijo et al., 2018). According to the spectral analyses (Figure 3G, H), the frequency band of 10–100 mHz played a crucial role in Ca^2+^-dependent neuritogenesis.

**Figure 4—figure supplement 1.**
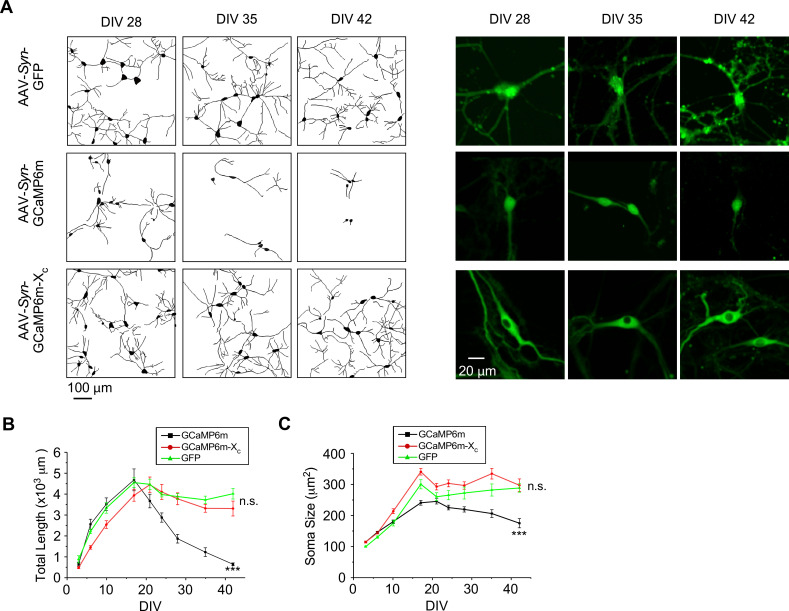
Indistinguishable neuronal morphology between GCaMP6m-X_C_ and GFP of long-term expression. (**A**) Representative neurite tracing of multiple neurons (left) and fluorescence images of individual neurons (right) for cultured cortical neurons expressing GCaMP6m-X_C_, GCaMP6m, or GFP on DIV 28, DIV 35, and DIV 42. Temporal profiles of the total length of neurites (**B**) and the soma size from DIV 3 to DIV 42 (**C**). Compared on DIV 42, no significant difference exists between GCaMP6m-Xc and GFP control; and the differences between GCaMP6m-Xc and GCaMP6m are significant. Three independent experiments from three independent culture preparations. Standard error of the mean (SEM) and one-way analysis of variance (ANOVA) followed by Bonferroni for post hoc tests (criteria of significance: ***p < 0.001; *n.s*. represents ‘not significant’) were calculated when applicable.

**Figure 4—figure supplement 2.**
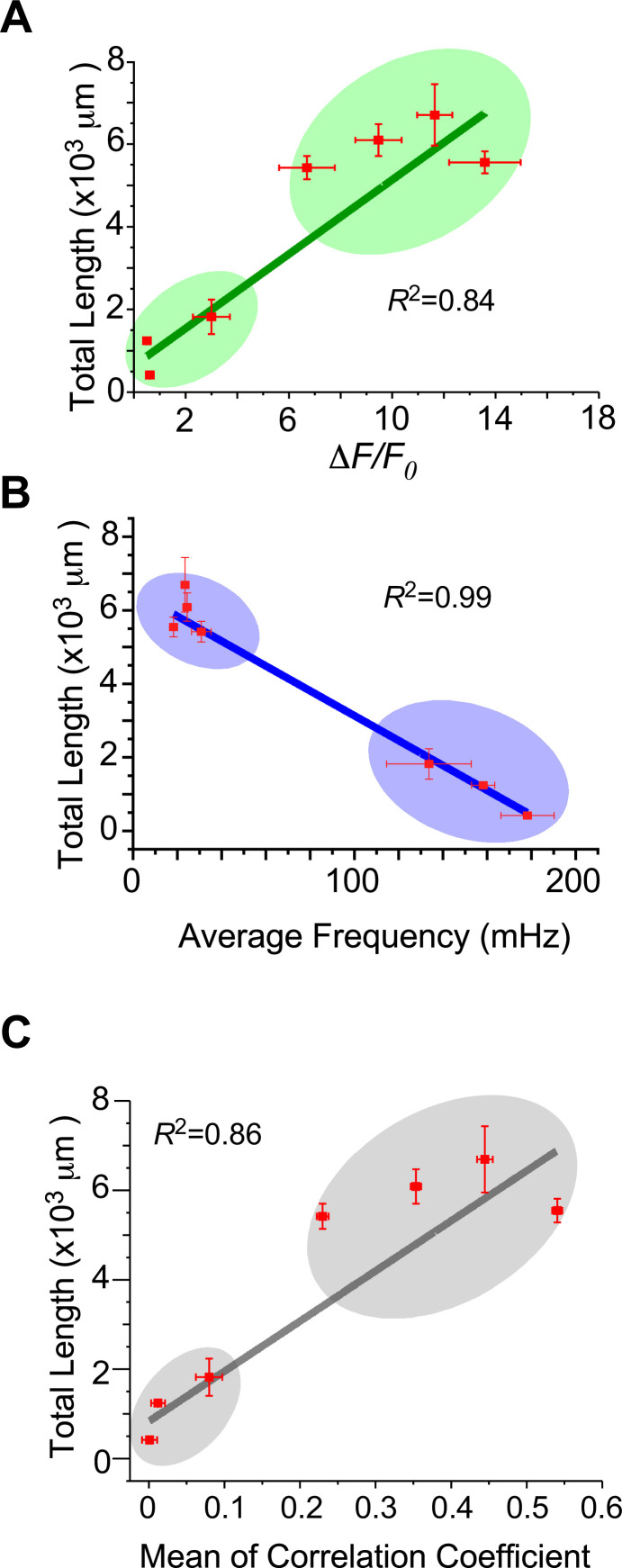
Potential relationships between and neurite length and oscillation characteristics. The peak amplitude (**A**), the average frequency (**B**), or the level of synchrony (**C**) was examined for its potential correlation with the total length of neurites, based on the data acquired by GCaMP6m-X_C_ in Figures 3 and 4. The linear correlation coefficient (*R*^2^) was evaluated between each index and neurite length. Standard error of the mean (SEM) was calculated when applicable.

**Figure 4—figure supplement 3.**
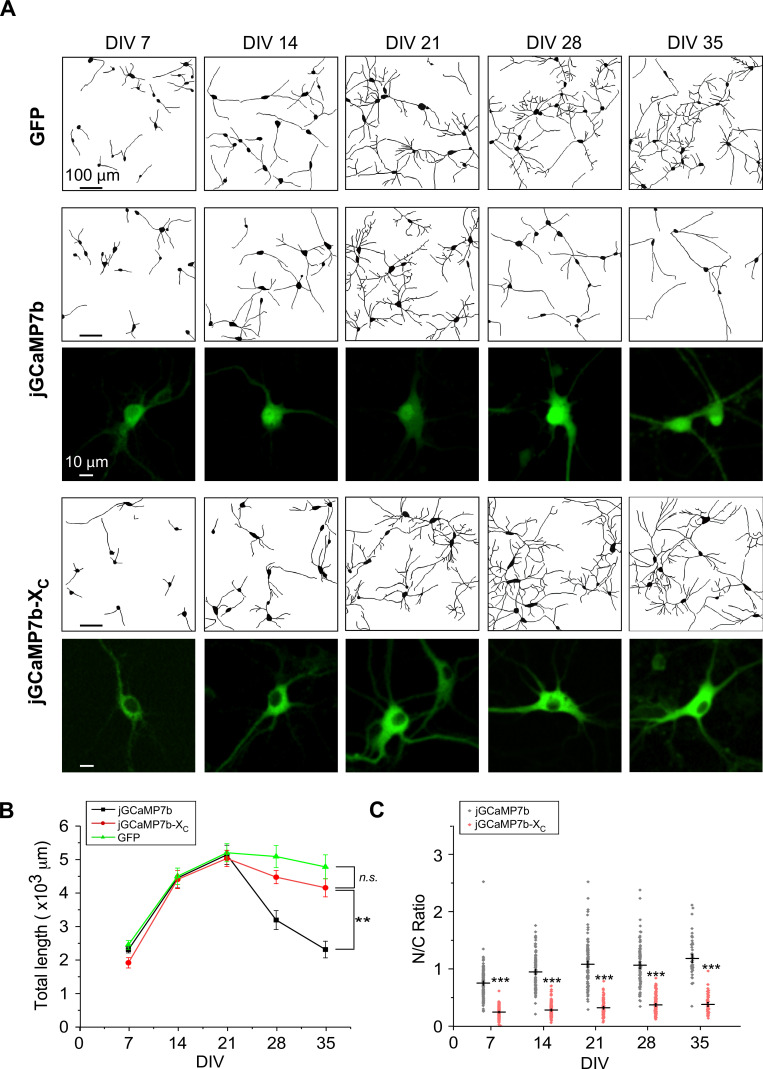
Morphological analysis to compare jGCaMP7b-X_C_ and jGCaMP7b in cultured cortical neurons. (**A**) Cortical neurons virally expressing jGCaMP7b-X_C_, jGCaMP7b, and GFP control, respectively. For each group, neurite tracing of multiple neurons (upper rows) and representative confocal fluorescence images of individual neurons (lower rows) at the selected timepoints of DIV 7 to DIV 35. (**B**) Temporal profiles of the total length of neurites summarized for jGCaMP7b-X_C_ and jGCaMP7b versus GFP control. (**C**) N/C ratio of neurons expressing jGCaMP7b or jGCaMP7b-X_C_. Three independent experiments from three independent culture preparations. Standard error of the mean (SEM) and one-way analysis of variance (ANOVA) followed by Bonferroni for post hoc tests (**B**) or two-tailed unpaired Student’s *t*-test (**C**) (criteria of significance: **p < 0.01; ***p < 0.001; *n.s*. represents ‘not significant’) were calculated.

**Figure 4—figure supplement 4.**
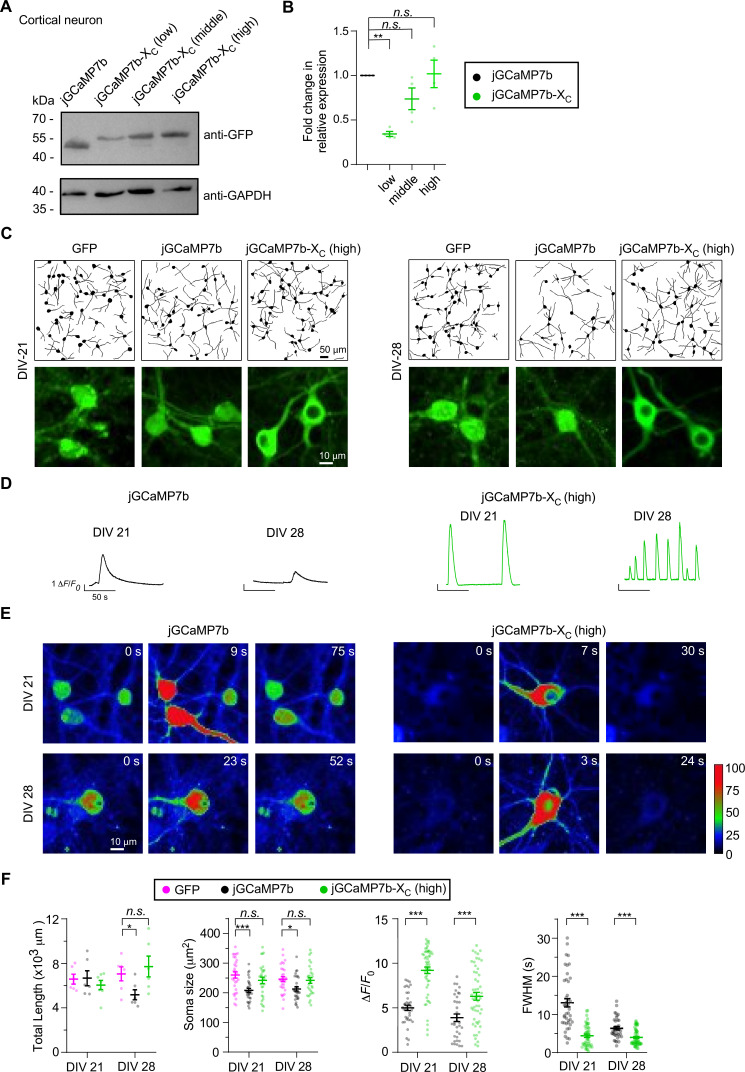
The long-term effects of GCaMP-X with enhanced expression levels in cultured neurons. (**A, B**) Protein expression levels in whole-cell extracts from cortical neurons infected with 1 μl AAV-*Syn*-jGCaMP7b (1.0 × 10^12^ v.g./ml) or 1 μl (low), 2 μl (middle), 3 μl (high) AAV-*Syn*-jGCaMP7b-X_C_ (1.0 × 10^12^ v.g./ml). By western blots, anti-GFP bands and anti-GAPDH bands indicated the level of probe expression and the amount of inputs, respectively. Statistical summary for whole-cell expression levels of low/middle/high-dose jGCaMP7b-X_C_ in comparison to jGCaMP7b. (**C–E**) Comparison between jGCaMP7b and high-dose jGCaMP7b-X_C_ in cultured cortical neurons. To compare neuritogenesis (**C**), neurons virally expressing GFP served as the control. Representative Ca^2+^ waveforms (**D**) and images (**E**) are shown for cultured cortical neurons virally expressing jGCaMP7b or jGCaMP7b-X_C_ (high) on DIV 21 and DIV 28. (**F**) Statistical summary of neurite growth and Ca^2+^ oscillation, to compare the three groups by the key indices including total neurite length per view, soma size of individual neurons, and peak amplitude and FWHM of Ca^2+^ waveform. Two independent experiments from two independent culture preparations. Standard error of the mean (SEM) and one-way analysis of variance (ANOVA) followed by Bonferroni for post hoc tests (criteria of significance: *p < 0.05; **p < 0.01; ***p < 0.001; *n.s*. represents ‘not significant’) were applied. Figure 4—figure supplement 4—source data 1.Source data for Figure 4—figure supplement 4A.Original uncropped western blotting gels with indication of the cropped areas.

Previous studies mainly relied on measuring transient Ca^2+^ and neurite growth rate within a brief period of time (Ito et al., 2010; Mukai et al., 2003; Rosenberg and Spitzer, 2011; van Pelt et al., 2004). However, the overall neurite outgrowth across the developmental phases may help elucidate the roles of Ca^2+^ in neuritogenesis, which has been lacking due to the difficulties of long-term Ca^2+^ imaging. In fact, contradictory observations have been reported regarding how Ca^2+^ actually regulates the rates of neuronal growth. Here, based on GCaMP-X imaging data, the first derivative of the growth curves was calculated as the growth rate of neurites or soma (Figure 4E). The bell-shaped curves suggested that the relationships between Ca^2+^ oscillations and neuronal growth rates may depend on developmental stages, which reached the peak rates around DIV 10 for both neurite length and soma size. Therefore, the maximum growth rate appeared to be determined by both amplitude and frequency of Ca^2+^ oscillations. In general, there might not be a simple relationship between oscillation characteristics and neuronal development. Within a short or brief timeframe, the growth rates in relation to various combinations of amplitude and frequency could be complicated (Gomez and Zheng, 2006), especially without considering the development stage of neurons. Similar results were obtained from jGCaMP7b-X_C_ in comparison with jGCaMP7b (Figure 4—figure supplement 3). In summary, chronic GCaMP-X imaging provided a glimpse of the potential roles of slow Ca^2+^ oscillations in neuritogenesis across multiple stages of neuronal development.

To further exclude any potential artifact related to probe expressions, a gradient of expression levels by jGCaMP7b-X_C_ viruses were examined in cultured cortical neurons (Figure 4—figure supplement 4). 3 μl AAV-*Syn*-jGCaMP7b-X_C_ (1.0 × 10^12^ v.g./ml) and 1 μl AAV-*Syn*-jGCaMP7b (1.0 × 10^12^ v.g./ml) led to the similar levels of whole-cell expression (the former would express much more in the cytosol). Under such conditions, the results from the two groups of neurons were consistent with those with equal amounts/volumes of viruses. jGCaMP7b-X_C_ was much less toxic than jGCaMP7b, by comparing the indices of neuronal growth and Ca^2+^ oscillations (Figure 4—figure supplement 4F), where neurite length and soma size of neurons expressing high-level jGCaMP7b-X_C_ were nearly indistinguishable from GFP control neurons.

### Chronic imaging of spontaneous Ca^2+^ activities in vivo

**Figure 5.**
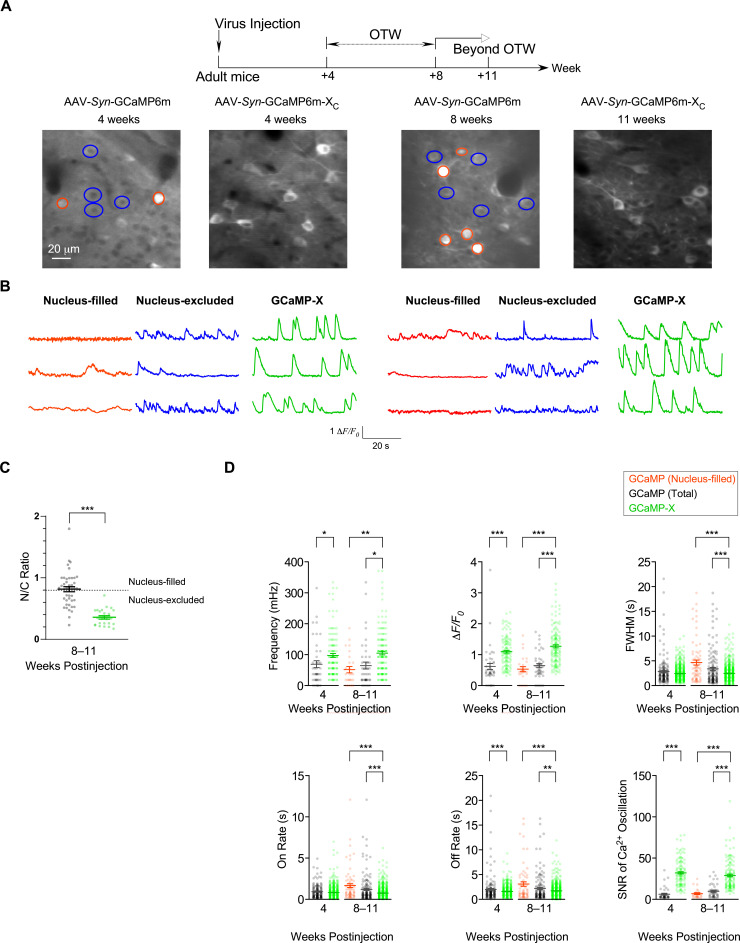
Chronic Ca^2+^ imaging for spontaneous Ca^2+^ activities in vivo. In vivo two-photon fluorescence images (**A**) and spontaneous nuclear Ca^2+^ activities (**B**) of virus-infected neurons in S1 primary somatosensory cortex. Neurons expressing GCaMP6m-X_C_ or GCaMP6m (nucleus-filled and nucleus-excluded) within OTW (optimal time window, 4–8 weeks postinjection) and beyond OTW (8–11 weeks postinjection) were analyzed and compared. (**C**) N/C ratio summary of GCaMP6m and GCaMP6m-X_C_ in vivo beyond OTW. Neurons were divided into two groups: nucleus-excluded and nucleus-filled, by applying the criteria of N/C ratio (0.8). (**D**) Key parameters of spontaneous Ca^2+^ activities. Standard error of the mean (SEM) and two-tailed unpaired Student’s *t*-test (**C**) or one-way analysis of variance (ANOVA) followed by Bonferroni for post hoc tests (**D**) (criteria of significance: *p < 0.05; **p < 0.01; ***p < 0.001) were calculated when applicable. Data were obtained from three or two mice for GCaMP6m and GCaMP6m-X_C_, respectively.

GCaMP6m perturbed autonomous Ca^2+^ oscillations, presumably as one leading cause of neuronal toxicities. Such tight correlations between Ca^2+^ dysregulations and aberrant morphology were clearly manifested during early development, which may extend onto mature neurons. The viruses were added to cultured cortical neurons at the mature stage (DIV 21), which were subsequently examined to compare the effects of jGCaMP7b versus jGCaMP7b-X_C_ (Figure 5—figure supplement 1). Analyses of both neurites and oscillations demonstrated similar side-effects of jGCaMP7b in comparison with jGCaMP7b-X_C_, starting to show up on DIV 28 and later on DIV 35 exhibiting significant differences in neurite length and oscillation characteristics. Similar to cultured neurons, spontaneous Ca^2+^ activities in vivo are also correlated to gene transcription and expression at the cellular and circuity levels (Laviv et al., 2020; Takahashi et al., 2016). Therefore, based on our experiments and other published reports, a common theme of correlation exists between spontaneous Ca^2+^ and neuronal morphology, for both premature and mature neurons, and both in vitro and in vivo (Figure 5—figure supplement 2). For adult mouse brain infected by AAV viruses of GCaMP6m or GCaMP6m-X_C_ (the same procedures and dosages as in Figure 2), we characterized spontaneous Ca^2+^ activities in S1 primary somatosensory cortex (Figure 5A, B and Figure 5—videos 1 and 2). Two checkpoints were set at 4 weeks postinjection (within OTW) and at 8 or 11 weeks postinjection (prolonged expression time beyond OTW), respectively. Similar to whisker deflection-response experiments in Figure 2, the nucleus-filled neurons exhibited noticeable abnormalities in spontaneous Ca^2+^ activities even within OTW. Beyond OTW, nucleus-filling was often found from GCaMP6m while very rare from GCaMP6m-X_C_ as indexed by N/C ratio (Figure 5C). Accordingly, the damage was much exacerbated, as evidenced from the total or nucleus-filled neurons expressing GCaMP in comparison with the total neurons expressing GCaMP-X (Figure 5D). With GCaMP6m, the frequency and amplitude resulted in significantly lower values, accompanied by aberrantly wider FWHM and slower on/off rates. In contrast, neurons expressing GCaMP6m-X_C_ maintained robust and stable spontaneous Ca^2+^ activities with key characteristics within the normal ranges across the full term of experiments (up to 11 weeks postinjection). Notably, GCaMP6m-X_C_ significantly improved the SNR calculated from spontaneous Ca^2+^ signals in vivo, both within and beyond OTW (Figure 5D).

**Figure 5—figure supplement 1.**
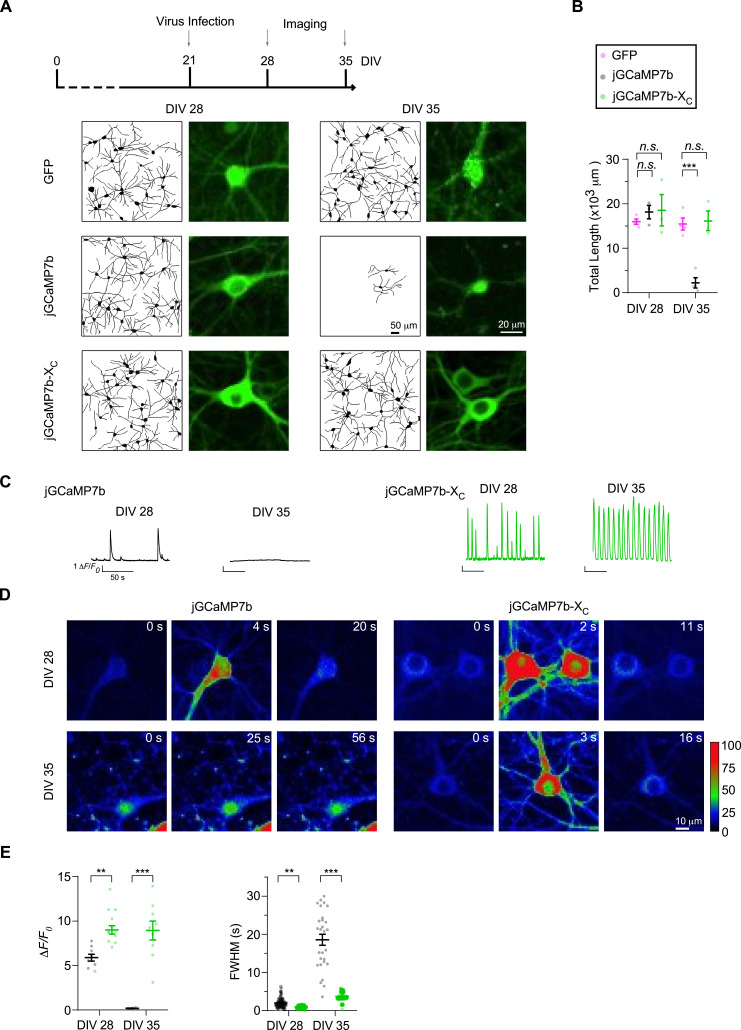
Effects of virally expressed GCaMP versus GCaMP-X on mature cortical neurons. (**A, B**) Cultured cortical neurons from newborn ICR mice were infected on DIV 21 with GFP, jGCaMP7b and jGCaMP7b-X_C_ viruses (2 μl of each), respectively. Then neurons were traced by confocal microscopy on DIV 28 and DIV 35 (**A**). Total length per view is summarized in (**B**). (**C, D**) Oscillatory Ca^2+^ waveforms associated with color-coded fluorescence images on DIV 28 and DIV 35. (**E**) The key indices with *ΔF*/*F*_*0*_ and FWHM measured from spontaneous Ca^2+^ in cultured neurons. Two independent experiments from two independent culture preparations. Standard error of the mean (SEM) and one-way analysis of variance (ANOVA) followed by Bonferroni for post hoc tests (**B**) or two-tailed unpaired Student’s *t*-test (**E**) (criteria of significance: **p < 0.01; ***p < 0.001; *n.s*. represents ‘not significant’) were calculated.

**Figure 5—figure supplement 2.**
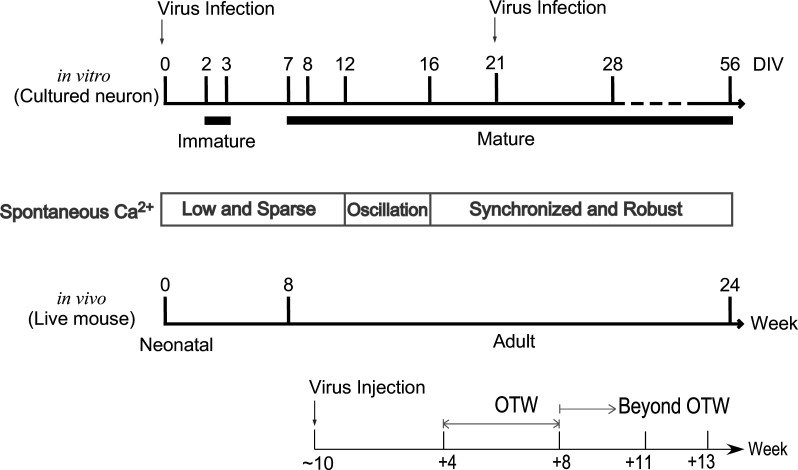
The critical timepoints of the protocols for both in vitro and in vivo experiments. Neurons at different timepoints exhibit diverse patterns of spontaneous Ca^2+^ fluctuations. In general, cultured neurons from neonatal mice around DIV 2–3 are considered to be immature with low and sparse oscillations (Isaev et al., 2018). Cultured neurons could enter into mature stage as early as ~DIV 7 (up to DIV 56), corresponding to 8- to 24-week-old adult mice in vivo (Dutta and Sengupta, 2016), where periodical oscillations from multiple neurons emerge around DIV 12–16. Robust and synchronized oscillations are observed usually after DIV 16 (Murphy et al., 2020; Opitz et al., 2002), which correspond to neural network formation and synaptic maturation (Mori and Mook-Jung, 2015). Virus infection was conducted for both neonatal (DIV 0) and mature (DIV 21) neurons to culture further for in vitro experiments. For in vivo studies, adult mice (>8 weeks) were used for virus injection. One to two months (4–8 weeks) postinjection and thereafter (8–13 weeks) are considered as within optimal time window (OTW) and beyond OTW, respectively (Chen et al., 2013; Resendez et al., 2016; Tian et al., 2009).

**Figure 5—video 1.** In vivo two-photon imaging of spontaneous Ca^2+^ oscillation of neurons virally expressing GCaMP6m-X_C_ in S1 primary somatosensory cortex beyond optimal time window (11 weeks postinjection). Enlarged images (512 × 512 pixels, 300 × 300 µm^2^) of L2/3 cells (150–250 µm under the pia) in S1BF were collected at 7.4 Hz frame rate. Video plays at 6× speed.

**Figure 5—video 2.** In vivo two-photon imaging of spontaneous Ca^2+^ oscillation of neurons virally expressing GCaMP6m in S1 primary somatosensory cortex beyond optimal time window (8 weeks postinjection). Enlarged images (512 × 512 pixels, 300 × 300 µm^2^) of L2/3 cells (150–250 µm under the pia) in S1BF were collected at 7.4 Hz frame rate. Neurons with nucleus-filled GCaMP6m barely fired. Video plays at 6× speed.

### Effects on neuronal morphology in vivo during long-term expression of GCaMP versus GCaMP-X

**Figure 6.**
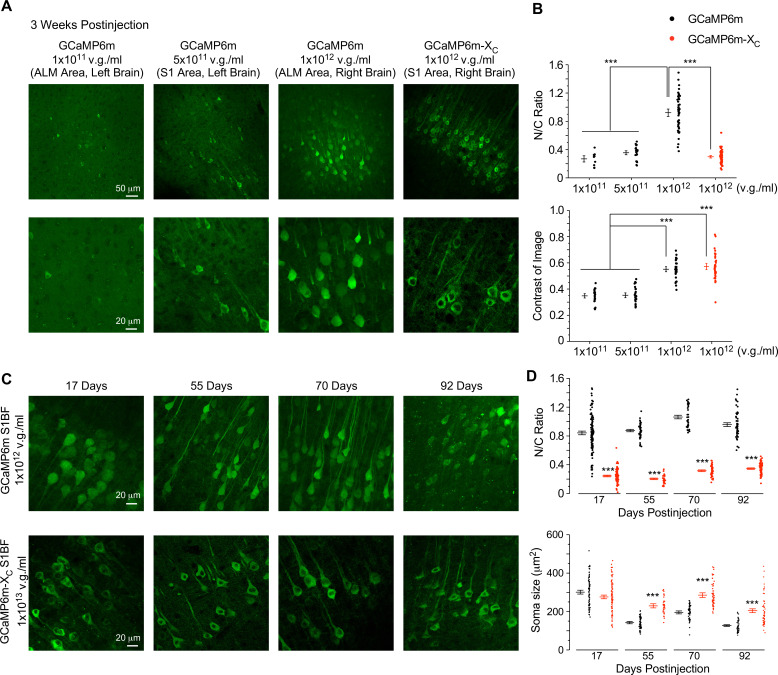
Evaluation of neuronal morphology for long-term in vivo expression of GCaMP-X versus GCaMP. (**A**) Confocal brain-slice images for ALM (anterolateral motor cortex) or S1 (primary somatosensory cortex). For the same mouse, AAV-*Syn*-GCaMP6m or AAV-*Syn*-GCaMP6m-X_C_ (30 nl) of the indicated titers were injected into the left or right brain. Brain slices were dissected 3 weeks after virus injection. (**B**) N/C ratio (upper) and contrast of images (lower), summarized over three mice. (**C**) Confocal images of brain slices expressing GCaMP6m or GCaMP6m-X_C_ acquired at different timepoints up to 92 days postinjection. AAV-*Syn*-GCaMP6m or AAV-*Syn*-GCaMP6m-X_C_ (60 nl) viruses of the indicated titers were microinjected into the left or right S1BF (barrel field of S1) of the same mouse, respectively. (**D**) Summary of N/C ratio (upper) and soma size (lower) for the neurons expressing GCaMP6m or GCaMP6m-X_C_ (eight mice in total). Standard error of the mean (SEM) and one-way analysis of variance (ANOVA) followed by Bonferroni for post hoc tests (criteria of significance: ***p < 0.001) were calculated when applicable.

Similar to chronic GCaMP-X imaging in vitro, a similar correlation has been expected between oscillatory Ca^2+^ and neuronal morphology for live adult mice which may underlie GCaMP side-effects observed from in vivo imaging (Figures 2 and 5). Firstly, titrations of GCaMP viruses were applied to characterize the dose-dependent damage of neurons, for which the concentrations were at 1 × 10^11^, 5 × 10^11^, and 1 × 10^12^ v.g./ml for AAV-*Syn*-GCaMP6m, and 1 × 10^12^ v.g./ml for AAV-*Syn*-GCaMP6m-X_C_, respectively. The viruses (30 nl) at the above concentrations were microinjected into different brain regions of the same mouse and then after 3 weeks the expression levels in brain slices were examined (Figure 6A). Low-concentration injection of virus at 1 × 10^11^ v.g./ml exhibited extremely sparse expression of GCaMP6m and yielded a low cell count. Correspondingly, the fluorescence signals were difficult to distinguish from the background, that is, low contrast and SNR. The virus concentration, when increased to 5 × 10^11^ v.g./ml, resulted in a relatively larger number of healthy-looking cells expressing GCaMP6m. But the low image contrast still affected proper detection of Ca^2+^ signals due to neuropil fluorescence. High-expression levels of GCaMP (at the virus concentration of 1 × 10^12^ v.g./ml) significantly enhanced the fluorescence image contrast and greatly increased the numbers of GCaMP-positive cells. However, the majority of neurons exhibited severe nuclear accumulation, which would subsequently lead to aberrant Ca^2+^ dynamics and cell death (Figure 6B). In contrast, high-dose injection of GCaMP6m-X_C_ virus at 1 × 10^12^ v.g./ml was beneficial for image contrast and the number of positive and healthy cells; meanwhile, the N/C ratio remained within the low range as expected. Next, under the conditions similar to Figures 2 and 5, we injected 60 nl GCaMP6m viruses of high dose (1 × 10^12^ v.g./ml) and GCaMP6m-X_C_ viruses of ultrahigh dose (1 × 10^13^ v.g./ml, 10-fold higher) to examine the temporal profile of damage in the same cortical region of S1BF (Figure 6C). At the checkpoints of 17-, 55-, 70-, and 92-day postinjection, neurons expressing GCaMP6m-X_C_ were compared with GCaMP6m. Confocal microscopy with brain slices revealed that the percentage of infected neurons and the expression level of GCaMP6m-X_C_ were close to their peaks on 17 days, suggesting that the ultrahigh dose could expedite GCaMP6m-X_C_ expression to reach the high level. Most importantly, long-term, high-level expression of GCaMP6m-X_C_ up to 92 days did not induce nuclear accumulation, whereas GCaMP6m at relatively lower concentration (1 × 10^12^ v.g./ml) already caused severe nuclear accumulation evidenced from 17 to 92 days (Figure 6D). Meanwhile, the soma size of GCaMP6m-infected neurons was significantly smaller than that of GCaMP6m-X_C_ from 55 to 92 days, presumably due to impaired spontaneous Ca^2+^ activities and related Ca^2+^ signals in these neurons. To directly confirm the relative expression levels, immunocytochemistry was performed on the brain slices infected by GCaMP6m and GCaMP6m-X_C_ viruses, under similar conditions as in Figure 6C, D. Long-term (13 weeks) expression levels of AAV-*Syn*-GCaMP6m (at the concentrations of 5 × 10^11^ and 1 × 10^12^ v.g./ml) in the brains of adult mice were quantified by anti-GFP immunofluorescence (Figure 6—figure supplement 1). And both dosages resulted in significantly lower expression levels than AAV-*Syn*-GCaMP6m-X_C_ of higher concentration (1 × 10^13^ v.g./ml), excluding the expression level as the cause of less damage. Of note, the soma size of neurons after long-term GCaMP-X expression was larger than GCaMP, while indistinguishable from the blank control neurons. In summary, in comparison with GCaMP, GCaMP-X exhibited high compatibility with neurons as desired by chronic Ca^2+^ imaging.

**Figure 6—figure supplement 1.**
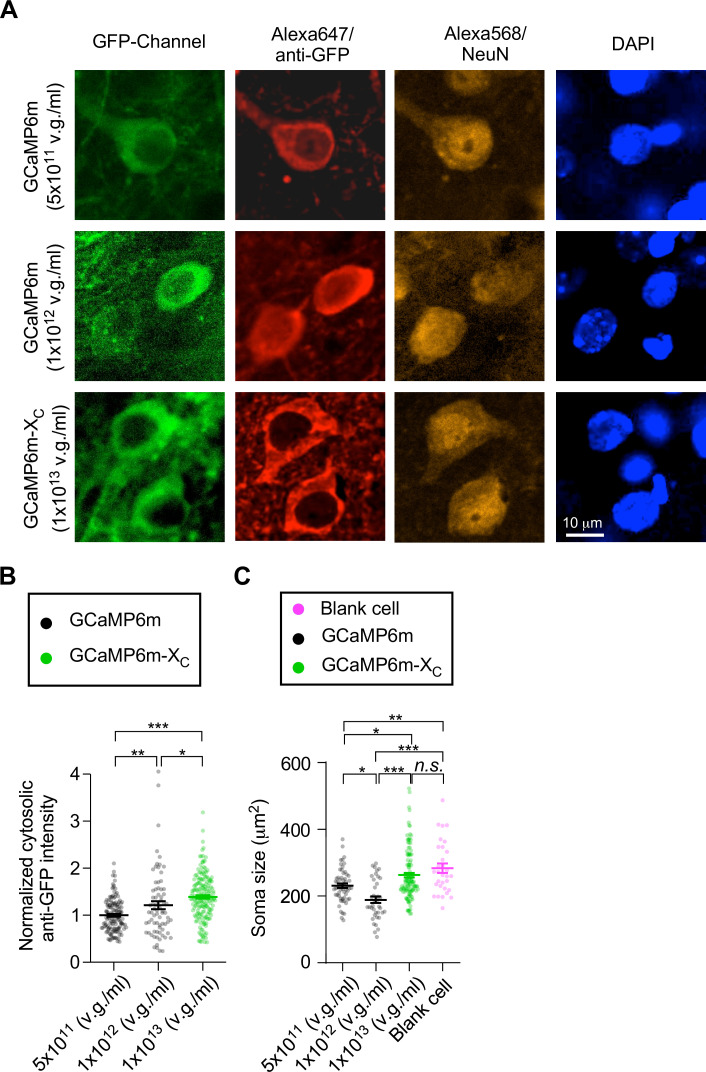
Long-term effects of GCaMP-X with high-expression levels in vivo. (**A**) Immunostaining images of neurons infected by AAV-*Syn*-GCaMP6m (5 × 10^11^ or 1.0 × 10^12^ v.g./ml, 60 nl) and AAV-*Syn*-GCaMP6m-X_C_ (1.0 × 10^13^ v.g./ml, 60 nl) at left or right S1BF. Thirteen weeks after microinjection, brain slices of injected mice were stained with anti-GFP (Alexa Fluo 647 nm, red), NeuN (Alexa Fluo 568 nm, yellow), and DAPI (blue) to reflect the protein expression of the indicators, cell bodies, and nuclear envelops, respectively. The GFP channel (green) was to indicate the positive neurons. Neurons without fluorescence in GFP channel and anti-GFP channel served as the blank cell control. (**B**) Cytosolic fluorescence intensity of anti-GFP of each neuron was normalized by that of low-dose GCaMP6m group (5 × 10^11^ v.g./ml) then summarized and compared. (**C**) Soma size calculated for each neuron and summarized to compare. The group of high-dose GCaMP6m-X_C_ had the highest level of expression whereas causing the least damage to neurons judged from its largest soma size among the three groups of neurons. Standard error of the mean (SEM) and one-way analysis of variance (ANOVA) followed by Bonferroni for post hoc tests (criteria of significance: *p < 0.05; **p < 0.01; ***p < 0.001; *n.s*. represents ‘not significant’) were applied.

### Transgenic GCaMP mice may have similar neuronal toxicities

Although the drawbacks of GCaMP were noticed at the very beginning of its invention and then improved by mechanism-based rational-design later on, GCaMP transgenic mice have been considered to be relatively safe in comparison with viral delivery of GCaMP. Nevertheless, recent studies reported that some transgenic mouse lines, such as Ai93 and Ai148, suffered from epileptiform activities (Daigle et al., 2018; Steinmetz et al., 2017). Based on our data thus far mostly by way of transient transfection and viral infection, we suspected that the mechanisms of side-effects are likely applicable to transgenic expression of GCaMP. Following up this hypothesis, brain slices and culture neurons from transgenic mice were examined from functional and morphological aspects.

**Figure 7.**
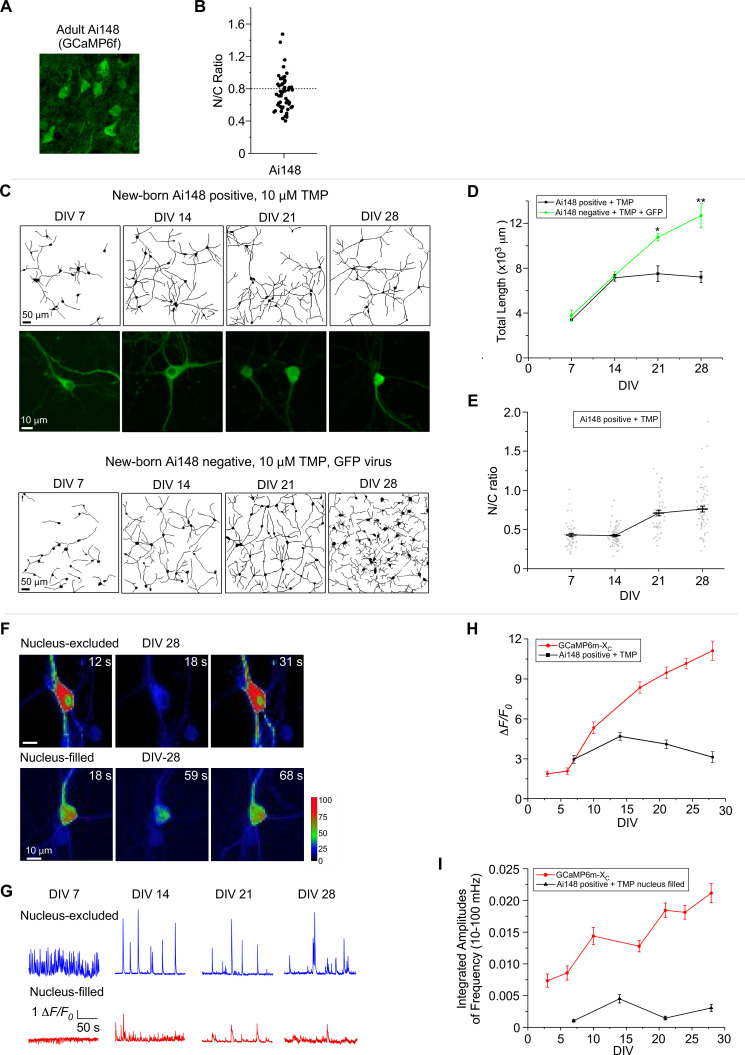
Chronic imaging for cultured cortical neurons of GCaMP transgenic mice. (**A**) Confocal brain-slice images of cortex layer II–III from 6-month-old GCaMP6f-positive transgenic mice (Rasgrf2-2A-dCre;Ai148 mice). GCaMP6f was examined ~4 months after induction of expression by intraperitoneal injection of TMP. (**B**) The N/C ratio of layer II–III neurons of adult Ai148 mice. (**C**) Neurite growth of cultured cortical neurons from newborn Ai148 mice. Neurite tracing for GCaMP-positive neurons (upper two rows) versus GCaMP-negative neurons (infected by GFP virus, bottom row), both added with 10 µM TMP to induce transgene expression. Zoomed confocal images illustrate subcellular distributions of GCaMP (middle row). Temporal profiles of total neurite length (**D**) or N/C ratio (**E**) for GCaMP-expressing Ai148 neurons, in comparison with GCaMP-negative GFP-infected neurons. (**F**) Time-laps images of single-neuron Ca^2+^ dynamics on DIV 28 from the nucleus-excluded and nucleus-filled subgroups. Color coding indicates fluorescence intensity. (**G**) Spontaneous Ca^2+^ activities of GCaMP-positive neurons in the nuclear-excluded and nuclear-filled subgroups from DIV 7 to DIV 28. Temporal profiles of the peak amplitudes (*ΔF*/*F*_*0*_, **H**) and the integrated amplitudes (over the frequency band of 10–100 mHz, **I**) for Ai148 neurons, compared with GCaMP-X_C_. Data were based on 3 Ai148 mice (**A, B**), and two independent experiments from two independent culture preparations (**C–I**). Standard error of the mean (SEM) and two-tailed unpaired Student’s *t*-test (**D**) (criteria of significance: *p < 0.05; **p < 0.01) were calculated when applicable.

Ai148 is a widely used transgenic line, for which TIGRE2.0 has been utilized for GCaMP6f to enhance its expression level, such that the damage by GCaMP is potentially more pronounced (Daigle et al., 2018). Using confocal fluorescence microscopy, we examined GCaMP-expressing neurons from the layer II–III cortex of the 6-month-old Rasgrf2-2A-dCre;Ai148 mice (with TMP-inducible expression of GCaMP6f) (Figure 7A). Nuclear accumulation of GCaMP was readily discernible, although it was relatively less severe than long-term expression of viral GCaMP6m of high doses (Figure 6). Next, dissected from newborn Rasgrf2-2A-dCre;Ai148 mice, cortical neurons were cultured, and subsequently 10 μM TMP was added to induce GCaMP expression. Similar to viral delivery, transgenic neurons were also subject to GCaMP6f perturbations, especially in nucleus-filled neurons. Neurite tracings indicated that the complexity and length of neurites were reduced in Ai148 neurons as compared to GFP control neurons from DIV 21 onwards (Figure 7C). The temporal profile of total neurite length indicated that neurite outgrowth was significantly slowed down or even halted on DIV 14, in comparison with the control neurons (GFP virus-infected and TMP treated) (Figure 7D). Consistently, N/C ratio of GCaMP indicative of nuclear accumulation was gradually increased along with the expression time up to 1 month (Figure 7E). Functionally, Ca^2+^ waveforms of lower amplitude were acquired from Ai148 neurons expressing nucleus-filled GCaMP across the full month than the nucleus-excluded subgroup (Figure 7F, G), consistent with the previous results by GCaMP plasmids and viruses that the side-effects would be exacerbated by nuclear GCaMP. Similar results were obtained from analyzing the peak amplitude and integrated frequency of Ca^2+^ oscillations by comparing nucleus-filled versus nucleus-excluded subgroups of neurons on DIV 14 or later (Figure 7H, I). Another trial of neurite tracing and Ca^2+^ imaging with Ai148 neurons confirmed the effects and analyses described above (Figure 7—figure supplement 1).

**Figure 7—figure supplement 1.**
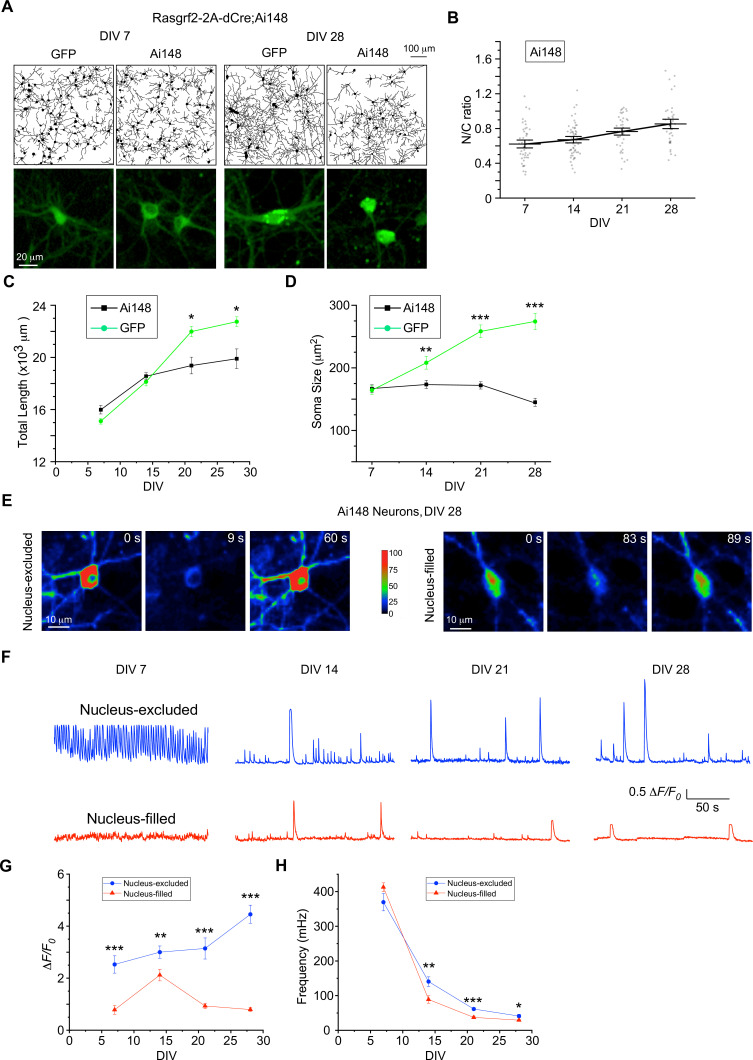
Chronic evaluation of cultured cortical neurons from Ai148 mice with inducible GCaMP6f expression. (**A**) Representative neurite tracing and fluorescence images for cultured cortical neurons expressing GCaMP6f or GFP on DIV 7 and DIV 28, respectively. GCaMP6f expression was induced by 10 μM TMP from Rasgrf2-2A-dCre;Ai148 mice. GFP control neurons were also treated with the same dose of TMP. Temporal profiles of N/C ratio (**B**), total neurite length (**C**), and soma size (**D**) for Ai148 neurons. (**E, F**) Spontaneous Ca^2+^ oscillations from the nucleus-excluded versus nucleus-filled (N/C ratio >0.8) subgroups of neurons from Ai148 mice. Time-laps color-coded Ca^2+^ fluorescence images on DIV 28 (**E**) and Ca^2+^ waveforms from DIV 7 to DIV 28 (**F**). Temporal profiles of peak amplitude (*ΔF*/*F*_*0*_, **G**) and oscillation frequency (mHz, **H**) Standard error of the mean (SEM) and Student’s *t*-test (two-tailed unpaired with criteria of significance: *p < 0.05; **p < 0.01; ***p < 0.001) were calculated when applicable.

**Figure 7—figure supplement 2.**
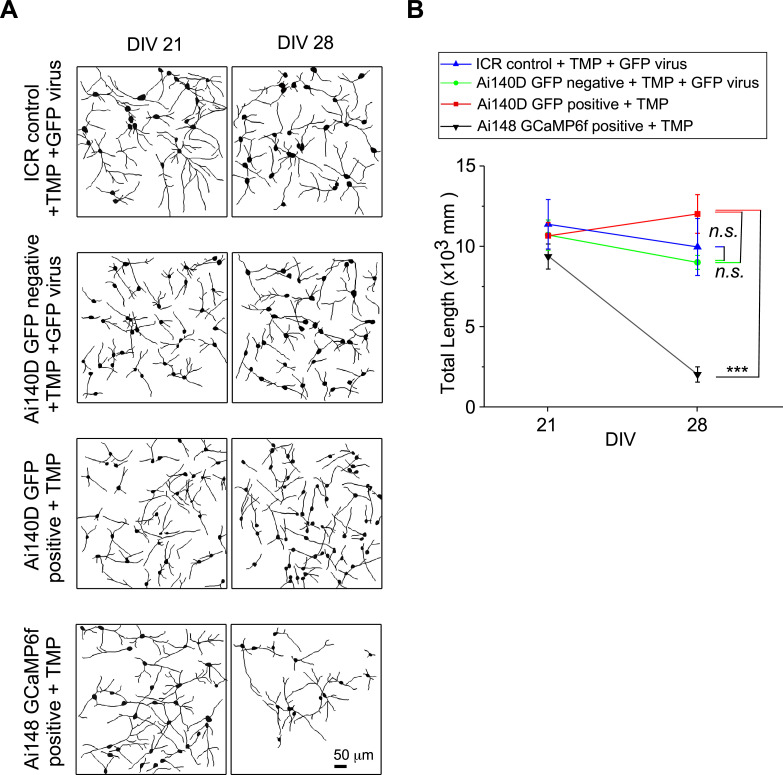
Neuritogenesis of cortical neuron from transgenic mice with TMP-inducible GCaMP. (**A**) Neurite tracings for Ai148 GCaMP6f-positive neurons (Rasgrf2-2A-dCre;Ai148 mice) versus Ai140D GFP-positive neurons (Rasgrf2-2A-dCre;Ai140D mice) on DIV 21 and DIV 28, both induced by 10 μM TMP. Ai140D GFP-negative neurons and ICR mouse neurons serves as additional controls, both infected GFP viruses, and also added with 10 μM TMP. (**B**) Statistical summary of total neurite length per view. No significant difference was found among ICR control, Ai140D GFP-negative, and Ai140D GFP-positive neurons, thus ruling out the potential biases or artifacts due to tTA toxicity, different strains of mice, or virus effects. The total length of neurites was significantly reduced in Ai148 GCaMP6f-positive neurons, as compared with Ai140D GFP-positive neurons. Standard error of the mean (SEM) and one-way analysis of variance (ANOVA) followed by Bonferroni for post hoc tests (criteria of significance: ***p < 0.001; *n.s*. represents ‘not significant’) were applied.

**Figure 7—figure supplement 3.**
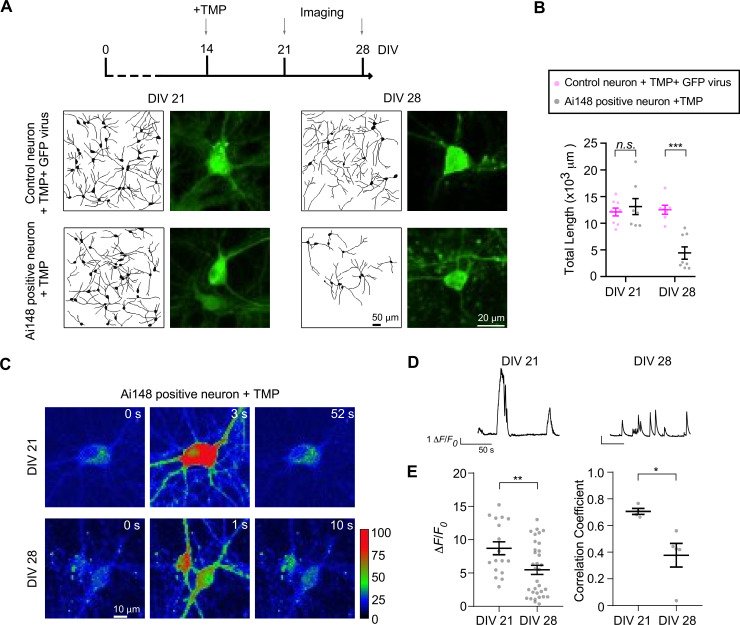
Effects on cortical neurons by transgenic GCaMP6f expression induced at the mature stage. (**A, B**) GCaMP6f was induced in cultured cortical neurons from Rasgrf2-2A-dCre;Ai148 mice by 10 μM TMP on DIV 14, then imaged by confocal microscopy on DIV 21 and DIV 28. Neurons from ICR mice treated with TMP treatment and infected by GFP virus served as the control. Based on neurite tracings (**A**), the total length per view (**B**) was summarized. Fluorescence images were zoomed in to show single neurons. (**C, D**) Oscillatory Ca^2+^ waveforms associated with color-coded fluorescence images on DIV 21 and DIV 28. (**E**) Statistical summary for the key indices of *ΔF*/*F*_*0*_ (from each neuron) and correlation coefficient (from each view). Two independent experiments were performed with two independent culture preparations. Standard error of the mean (SEM) and two-tailed unpaired Student’s *t*-test (criteria of significance: *p < 0.05; **p < 0.01; ***p < 0.001; *n.s*. represents ‘not significant’) were applied.

**Figure 7—figure supplement 4.**
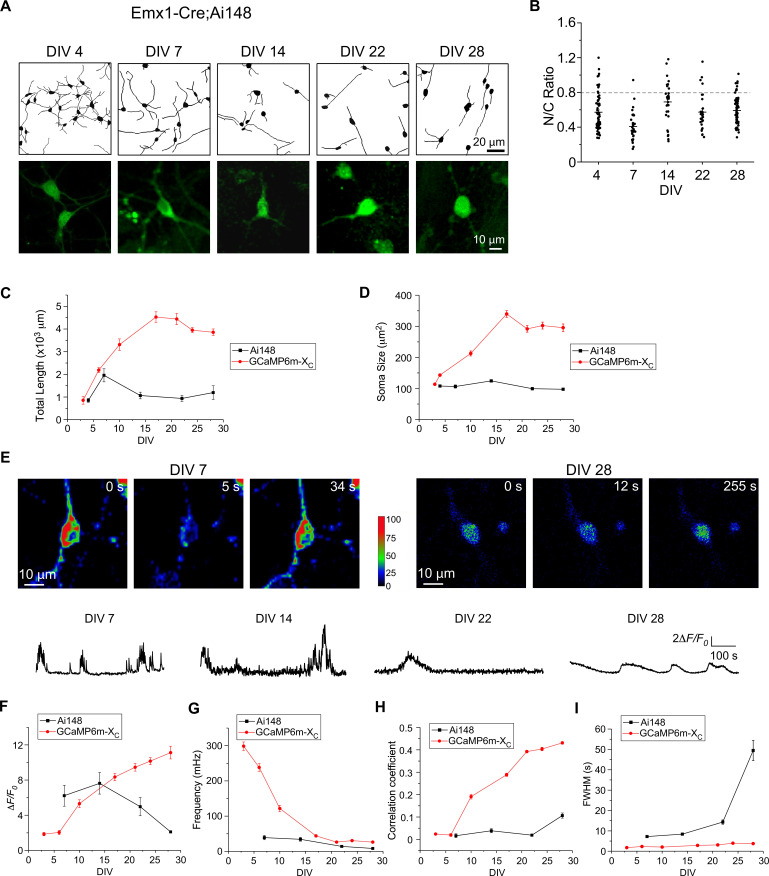
Long-term culturing and imaging of cortical neurons from Emx1-Cre;Ai148 mice with constitutive GCaMP6f expression. (**A**) Representative neurite tracing and fluorescence images for cultured cortical neurons constitutively expressing GCaMP6f from Emx1-Cre;Ai148 mice on selected DIV. (**B**) Temporal profiles of N/C ratio collected from Ai148 neurons. The dotted line indicates the criteria of N/C ratio 0.8. (**C, D**) Temporal profiles of total neurite length (**C**) and soma size (**D**). Data points and lines in red represent the average results (over 3 independent trials of ~4-week culturing/imaging) from viral GCaMP6m-X_C_. (**E**) Oscillatory Ca^2+^ waveforms associated with color-coded fluorescence images. (**F-I**) Temporal profiles of peak amplitude (*ΔF/F_0_*, **F**), oscillation frequency (mHz, **G**), synchronization (correlation coefficient, **H**), and FWHM (sec, **I**). Data points and lines in red represent the average results (over 3 independent trials of ~4-week culturing/imaging) from viral GCaMP6m-X_C_. Standard error of the mean (SEM) was calculated when applicable.

Comparing with GFP control neurons from Ai140D mice, the potential effects of tTA (Moullan et al., 2015) were excluded from the major results and conclusions regarding GCaMP toxicities (Figure 7—figure supplement 2). Also, by adding TMP on DIV 14 to induce transgenic GCaMP6f expression at the mature stage, similar damage on neurite morphology and Ca^2+^ oscillation was observed from Ai148 neurons, consistent with the previous notions that both premature and mature neurons are subject to GCaMP perturbations (Figure 7—figure supplement 3). In addition to chemical-inducible expression of GCaMP, newborn Emx1-Cre;Ai148 mice were deployed to constitutively express GCaMP6f (Figure 7—figure supplement 4). GCaMP6f started to accumulate in the nucleus at very early stage indexed by N/C ratio (criteria of 0.8). The damage was clearly evidenced when compared with the control neurons virally expressing GCaMP6m-X_C_. Meanwhile, the major characteristics of spontaneous Ca^2+^ oscillation in transgenic neurons were also significantly altered, resulting in relatively lower frequency, less synchronization, smaller amplitude, and abnormally wider FWHM.

In summary, the major findings by virus-infected neurons are applicable to transgenic mice, where GCaMP expression is either TMP inducible or constitutive. Morphological and functional analyses strongly suggest that cortical neurons in transgenic GCaMP6 mice are also subject to GCaMP toxicity similar to virus-infected neurons.

## Discussion

In this work, we applied GCaMP-X with reduced cell toxicity or enhanced neuron compatibility, to monitor Ca^2+^ dynamics across multiple days/weeks both in vitro and in vivo. By way of transient transfection, viral infection or transgenic expression, GCaMP of prolonged/excessive expression caused neuronal toxicities presumably due to its perturbations on endogenous apoCaM interactions, which were significantly reduced by rationally designed GCaMP-X. By relieving the concerns on the time and level of probe expression, GCaMP-X provides a simple solution for chronic calcium imaging in alternative to circumventing GCaMP toxicity. GCaMP-X paves the way to unexplored directions previously impeded or discouraged due to GCaMP perturbations.

### Available design solutions to avoid the side-effects of CaM-based GECI

To better utilize CaM-based GECIs in vitro and in vivo, solutions with no or minimum side-effects are in need. In utero electroporation and viral infection often result in high-expression levels particularly near the injection site, and some lines of transgenic mice using weaker promoters could control probe expression within the low levels to alleviate nuclear accumulation (Akerboom et al., 2012; Dana et al., 2019). Since probes present in the cytosol are able to bind apoCaM targets including Ca_V_1 channels (Yang et al., 2018), neurons may still suffer from the toxicity of the probes. Evidently, cytosolic GCaMP affected neural excitability in transgenic mice expressing GCaMP5G or GCaMP6 (Steinmetz et al., 2017). To overcome these problems, one solution is to substitute the core components of GECI design, for example, to utilize troponin C from muscle as a Ca^2+^-binding motif (Mank et al., 2008). The TN-XXL has been claimed to be suitable for chronic imaging potentially benefitted from its design basis (less likely to bind endogenous proteins in neurons). However, the TN-XXL solution has at least two shortcomings. First, TNXXL is a FRET-based ratiometric sensor, of which the dynamic range is limited by FRET methods and indeed much narrower than GCaMP. Second, the Ca^2+^-binding motif from mammalian troponin C has the canonical EF-hands (resembling CaM), thus still possible to perturb neurons by binding endogenous targets at the apo states, which needs further investigations.

The approach adopted by this work is to introduce an additional protective motif that specifically binds apoCaM within the probe (Figure 1A). Such CBM has been fused onto the N-terminus of conventional GCaMPs (from GCaMP3 to jGCaMP7) to construct a new series of GCaMP-X correspondingly. When Ca^2+^ level is low, CBM successfully prevents apoCaM contained within GCaMP-X from interfering with Ca_V_1 channels and other important apoCaM targets (Figure 1—figure supplement 3). Once Ca^2+^ concentration rises, M13 binds to Ca^2+^/CaM with high affinity, without altering Ca^2+^-sensing characteristics of GCaMP-X inherited from years of efforts and improvements. With GCaMP-X as the proof-of-principle, the design rules centered with apoCaM/Ca^2+^-CaM binding are potentially applicable to CaM-based sensors or actuators of a broader scope (Grødem et al., 2021; Haiech et al., 2019), which may face similar challenges or problems to those associated with enhanced/prolonged expression of GCaMP.

### Spontaneous Ca^2+^ activities in association with neural development and degeneration

While the membrane voltage is oscillating, cellular Ca^2+^ signals are also fluctuating, closely involved in neuronal development and circuit formation both in intro and in vivo (Kirkby et al., 2013; Sun et al., 2010). Meanwhile, in line with the aforementioned calcium hypothesis, dysregulated spontaneous Ca^2+^ activities would lead to defective morphology and functions of neurons, and eventually neural diseases (Harr and Distelhorst, 2010; Khan et al., 2020; Nicotera and Orrenius, 1998). In Ca^2+^ imaging experiments, Ca^2+^ fluorescence signals and electrical activities are often referred to each other since action potentials initiated by Na^+^ channels would subsequently drive the fast opening of Ca^2+^ channels that mediate Ca^2+^ influx. Electrical recording via MEA (multiple electrode array) has been widely applied in long-term brain/neuron monitoring (Obien and Frey, 2019; Shafer, 2019), among other methods. On the other hand, both membrane potentials and ion fluxes (Na^+^ or Ca^2+^) could have sophisticated mechanisms and specific consequences, for example, Ca^2+^ oscillations of different forms: subthreshold oscillations by L-type Ca^2+^ channels, or intracellular Ca^2+^ fluctuations by intracellular Ca^2+^-release channels (Chan et al., 2007; Uhlén and Fritz, 2010). GECIs are promising tools to overcome the limitations of many other methods including MEA, if the cell compatibility issues could be resolved as demonstrated by GCaMP-X in this work. GECI imaging methods directly and faithfully capture Ca^2+^ activities at different loci in the brain or within the cell, allowing high spatial–temporal resolution of concurrent morphological/functional imaging. Subcellular Ca^2+^ oscillations may be responsible for different aspects of neurogenesis and neuritogenesis, awaiting future investigations with organelle-specific GCaMP-X, such as the nuclear version GCaMP-X_N_. Earlier Ca^2+^ activities (weak fluctuations of higher frequency) may represent spontaneous activity before synapse or network formation (Spitzer, 2006). At the later stage, synchronized Ca^2+^ oscillations (of lower frequency) emerge, along with dramatic changes in morphology and other aspects of development. Autonomous oscillations of SNc neurons are tightly coupled with Ca_V_1.3 channels which may underpin Parkinson’s disease or neural aging (Chan et al., 2007; Guzman et al., 2009). Cultured cortical slices and hiPSC-derived cortical neurons also suggest that L-type Ca^2+^ channels are crucial for both spontaneous Ca^2+^ activities and neuronal development (Horigane et al., 2021; Plumbly et al., 2019). A similar mechanism is likely shared by the correlation between spontaneous/oscillatory Ca^2+^ activities and neuronal morphology/development unveiled in this study. Expression, trafficking and functions of ion channels and membrane receptors are also subject to regulations by activities of different patterns (Ruffinatti et al., 2013; Spitzer, 2006; Toth et al., 2016), and chronic GECI imaging is expected to help elucidate these compound effects and mechanisms. In this work, we have particularly focused on spontaneous Ca^2+^ activities of cortical neurons in association with neuronal morphology both in vitro (Figures 3 and 4) and in vivo (Figures 2, 5 and 6), and in both neonatal (Figures 3 and 4) and mature neurons (Figure 5—figure supplement 1 and Figure 7—figure supplement 3), as the exemplars to demonstrate the performance of chronic GCaMP-X imaging. Importantly, since such Ca^2+^ activity-neuronal morphology coupling was perturbed by GCaMP under various testing conditions, we are expecting a broad scope of applications awaiting GCaMP-X to explore both in vitro and in vivo.

### Improved neuron compatibility of GCaMP-X

GCaMP, as widely applied GECI, has been evolved into its eighth version (Grødem et al., 2021), with enhanced sensitivity, brightness and kinetic properties tailored to specific imaging purposes, including single action potentials and activities in neuronal populations and microcompartments (Dana et al., 2019). However, the cytotoxicity of GCaMP has been a persistent problem from early on, mainly due to the fact that the CaM-centered schemes of GCaMP have been largely inherited across generations of its design (Akerboom et al., 2012; Chen et al., 2013; Dana et al., 2019; Nakai et al., 2001; Tallini et al., 2006; Tian et al., 2009). In vivo Ca^2+^ imaging with GCaMP viruses is facing the dilemma of safety versus reliability. On the one hand, reducing GCaMP levels could alleviate or postpone the cytotoxicity, but low levels of expression would also reduce the contrast and SNR (Rose et al., 2014). On the other hand, increasing the expression level of GCaMP would enhance the data quality of Ca^2+^ imaging, but exacerbate the damage to neurons (Resendez et al., 2016). It is not surprising that GCaMP transgenic mice have encountered with similar problems. Several lines of GCaMP transgenic mice reported earlier, such as Emx1-Cre;Ai38 GCaMP3 transgenic mice, attempted to resolve the safety issue by restricting the expression to ultra-low levels (~5 μM) (Zariwala et al., 2012), but sacrificing the imaging quality (Rose et al., 2014). The mouse lines reported recently, such as Emx1-Cre;CaMK2α-tTA;Ai93 GCaMP6f transgenic mice and Slc17a7-IRES2-Cre;Ai148 GCaMP6f transgenic mice (Daigle et al., 2018; Madisen et al., 2015), managed to elevate the expression levels. However, epileptiform activities have been observed from these mice presumably due to GCaMP perturbations (Daigle et al., 2018; Steinmetz et al., 2017). One bypass solution is to conditionally induce GCaMP expression to conduct GCaMP imaging within a time window, which would be much less feasible for long-term expression and/or chronic imaging.

Instead, GCaMP-X allows long-term and high-level expression to increase the quality and reliability of Ca^2+^ imaging while reducing neuronal toxicities. For in vitro studies, investigations of long-term Ca^2+^ dynamics are largely hindered by the cytotoxicity of GECIs or dyes (Rose et al., 2014; Smith et al., 2018). GCaMP-X is well suited for longitudinal Ca^2+^ dynamics, for example, during neural development as in this study, for high-throughput screening of long-term drug effects (Vetter et al., 2020), or in other similar scenarios. For in vivo studies, due to the concerns known to GCaMP, false-negative or false-positive results by nuclear-filled GCaMP or under-expressed GCaMP are misleading especially in imaging large populations of neurons (Resendez et al., 2016). The existing reports have not yet reached an agreement regarding whether nuclear GCaMP would cause neuronal toxicities with a significant impact during the early postinjection phase, for example, Figures 2 and 5. Such discrepancy may reflect different doses of virus injection, different expression levels of GCaMP or even different types of neurons in different brain regions. Alternatively, it may still reinforce that neuronal damage could still exist even within OTW, as a precaution for selecting neurons and planning experiments. In this regard, GCaMP-X provides a viable option with higher SNR, more healthy neurons, lower neurotoxicity, prolonged expression/imaging, and meanwhile less experimental complexity.

Additional control experiments would help evaluate how close GCaMP-X data are to the reality, considering potential Ca^2+^-buffering effect intrinsic to Ca^2+^ probes and also other factors. Applicable controls were incorporated to better evaluate GCaMP-X data, for example, Ai140D mice (GFP, Figure 7—figure supplement 2) and Fluo-4 AM (Ca^2+^ dye, Figure 3—figure supplement 4). The results have been encouraging in that GCaMP-X neurons were nearly indistinguishable in the morphological and functional aspects from Ai140D neurons expressing GFP or loaded with Fluo-4 AM. The feedbacks from GCaMP-X applications should continue to help clarify this matter in the future.

## Materials and methods

**Key resources table:** 

Reagent type (species) or resource	Designation	Source or reference	Identifiers	Additional information
Strain, strain background (*Mus musculus*)	ICR	Institute of Cancer Research		Produced by Tsinghua University & Beihang University
Strain, strain background (*Mus musculus*)	C57	Jackson Labs	C57BL/6J; stock no. 000664	Produced by Tsinghua University
Strain, strain background (*Mus musculus*)	Ai148 (or Ai148D)	Jackson Labs	B6.Cg-Igs7tm148.1(tetO-GCaMP6f,CAG-tTA2)Hze/J; stock no.030328	
Strain, strain background (*Mus musculus*)	Rasgrf2-2A-dCre	Jackson Labs	B6;129S-Rasgrf2tm1(cre/folA)Hze/J; stock no.022864	
Strain, strain background (*Mus musculus*)	Ai140D	Jackson Labs	B6.Cg-Igs7tm140.1(tetO-EGFP,CAG-tTA2)Hze/J; stock no.030220	
Strain, strain background (*Mus musculus*)	Emx1-cre	Jackson Labs	B6.129S2-Emx1tm1(cre)Krj/J; stock no.005628	
Cell line (*Homo sapiens*)	Kidney (normal epithelial, embryo)	ATCC	HEK293	
Antibody	anti-GFP antibody (Rabbit polyclonal)	Abcam	Cat#ab290; RRID: AB_2313768	WB (1:1000); IF (1:200)
Antibody	anti-GAPDH antibody (Rabbit polyclonal)	Gene-Protein Link	Cat#P01L081	WB (1:10,000)
Antibody	anti-Histone H3 antibody (Mouse monoclonal)	Beyotime	Cat#AF0009; RRID: AB_2715593	WB (1:1000)
Antibody	anti-Calmodulin 1/2/3 antibody (Rabbit monoclonal)	Abcam	Cat#ab45689; RRID: AB_725815	WB (1:10,000)
Antibody	anti-His antibody (Rabbit monoclonal)	Gene-Protein Link	Cat#P01L071	WB (1:2000)
Antibody	HRP-conjugated Affinipure Goat Anti-Mouse IgG (H + L)	Proteintech	Cat#SA00001-1; RRID: AB_2722565	WB (1:10,000)
Antibody	HRP-conjugated Affinipure Goat Anti-Rabbit IgG (H + L)	Proteintech	Cat#SA00001-2; RRID: AB_2722564	WB (1:10,000)
Antibody	anti-NeuN antibody (Mouse monoclonal)	Millipore	Cat#MAB377; RRID: AB_2298772	IF (1:500)
Antibody	Goat anti-Mouse IgG (H+L) Alexa Fluor 568 (Goat polyclonal)	Invitrogen	Cat#A11004; RRID:AB_2534072	IF (1:800)
Antibody	Goat anti-Rabbit IgG (H+L) Alexa Fluor 647 (Goat polyclonal)	Invitrogen	Cat#A21244; RRID:AB_2535812	IF (1:800)
Chemical compound, drug	DAPI	Beyotime	2-(4-Amidinophenyl)-6-indolecarbamidine dihydrochloride; Cat#C1002	IF (1:1000)
Chemical compound, drug	TMP	Sigma	Trimethoprim, Cat#T7883-5G	Dilute to DMSO
Chemical compound, drug	Fluo-4 AM	Beyotime	Cat#S1060	
Chemical compound, drug	Pluronic F-127	Beyotime	Cat#ST501-1g	
Commercial assay or kit	Nuclear extraction kit	Abcam	Cat#113474	
Software, algorithm	GraphPad Prism v8.0.1	GraphPad Software	RRID:SCR_002798	
Software, algorithm	Imaris Viewer x64 v7.7.2	Oxford Instruments Group	RRID:SCR_007370	
Software, algorithm	Fiji v6.6.1	National Institutes of Health	RRID:SCR_002285	
Software, algorithm	Origin 2019b	OriginLab	RRID:SCR_014212	
Software, algorithm	Clampex	Molecular Devices	RRID:SCR_011323	
Software, algorithm	Matlab	MathWorks	RRID:SCR_001622	
Recombinant DNA reagent	pGP-CMV-GCaMP6m (plasmid)	PMID:23868258	RRID:Addgene_40754	
Recombinant DNA reagent	pEGFP-N1-GCaMP6m-X_C_ (plasmid)	PMID:29666364	RRID:Addgene_111543	
Recombinant DNA reagent	pGP-CMV-jGCaMP7b (plasmid)	PMID:31209382	RRID:Addgene_104484	
Recombinant DNA reagent	pEGFP-N1-jGCaMP7b-X_C_ (plasmid)	Created in this study	RRID:Addgene_178361	NES tag added to jGCaMP7b-X，generated by Liu Lab.
Recombinant DNA reagent	pEGFP-N1-jGCaMP7b-X_N_ (plasmid)	Created in this study	RRID:Addgene_178362	NLS tag added to jGCaMP7b-X，generated by Liu Lab.
Recombinant DNA reagent	pcDNA3-α_1DL_ (plasmid)	PMID:35589958		Plasmid to express full length α_1DL_，generated by Liu Lab.
Recombinant DNA reagent	pcDNA3-α_1DL_-3xFlag (plasmid)	PMID:35589958		Plasmid to express full length α_1DL_ with C-terminal 3xFlag tag，generated by Liu Lab.
Recombinant DNA reagent	pcDNA3-Flag-CaMBD_α_1DL_ (plasmid)	Created in this study		Plasmid to express CaMBD of α_1DL_ with N-terminal Flag tag，generated by Liu Lab.
Recombinant DNA reagent	pcDNA3.1-YFP-Ng_S36A-Myc-HisA (plasmid)	Created in this study		Plasmid to express Ng_S36A with C-terminal Myc-His tag，generated by Liu Lab.
Other	AAV2/DJ-*Syn*-GCaMP6m	Hanbio Biotechnology		Adeno-associated virus to express GCaMP6m in vitro
Other	AAV2/DJ-*Syn*-GCaMP6m-X_C_	Hanbio Biotechnology		Adeno-associated virus to express GCaMP6m-X_C_ in vitro
Other	AAV2/DJ-*Syn*-jGCaMP7b	Hanbio Biotechnology		Adeno-associated virus to express jGCaMP7b in vitro
Other	AAV2/DJ-*Syn*-jGCaMP7b-X_C_	Hanbio Biotechnology		Adeno-associated virus to express jGCaMP7b-X_C_ in vitro
Other	AAV2/DJ-*Syn*-ZsGreen	Hanbio Biotechnology		Adeno-associated virus to express ZsGreen in vitro
Other	pLenti-*Syn*-mCherry	OBiO Technology		pLenti virus to express mCherry in vitro
Other	AAV2/9-*Syn*-GCaMP6m	BrainVTA		Adeno-associated virus to express GCaMP6m in vivo
Other	AAV2/9-*Syn*-GCaMP6m-X_C_	BrainVTA		Adeno-associated virus to express GCaMP6m-X_C_ in vivo

### Molecular biology

The plasmids of jGCaMP7b-X_C_ and jGCaMP7b-X_N_ were constructed by replacing previously reported GCaMP6m-X_C_ or GCaMP6m-X_N_ (Yang et al., 2018) with appropriate PCR-amplified segments from jGCaMP7b via unique EcoRI and HindIII sites, or EcoRI and NotI sites, respectively. pcDNA3-Flag-CaMBD_α_1DL_ was generated by replacing YFP from pcDNA3-YFP-preIQ_3_-IQ_D_-PCRD_D_ (Yang et al., 2022), with PCR-amplified Flag (DYKDDDDK) to by KpnI and NotI. pcDNA3.1-YFP-Ng_S36A-Myc-His was generated by inserting the PCR-amplified segments of neurogranin (NM_024140.2) containing S36A into a customized pcDNA3.1-MCS-Myc-His vector via unique EcoRI and HindIII sites.

### Mice

Procedures involving animals have been approved by local institutional ethical committees (IACUC in Tsinghua University and Beihang University), similar to the previous protocol (Huber et al., 2012). In vivo experiments were conducted with adult mice (C57BL/6J, both male and female) of 2–6 months old. In total, 23 mice (C57BL/6J) were used for expression tests and functional tests (GCaMP6m and GCaMP6m-X_C_). Three mice of Ai148;Rasgrf2-2A-dCre (Jax #030328; Jax #022864) were used for brain-slice imaging to examine transgenic GCaM6f neurons. Expression of GCaMP6f in Ai148;Rasgrf2-2A-dCre mice was induced with antibiotic Trimethoprim (TMP) by intraperitoneal injection with the dose of 0.25–0.5 mg/g in vivo (Sando et al., 2013). In vitro experiments were based on data from 82 mice (P0–P1, both male and female). Fifty-two ICR mice were used for expression and functional tests of Ca^2+^ indicators. Two newborn Ai148;Emx1-Cre mice were used to persistently express GCaMP in cultured neurons. In the tests of cortical neurons expressing GCaMP6f from Ai148;Rasgrf2-2A-dCre mice, 15 Ai148; Rasgrf2-2A-dCre GCaMP6f-positive mice were compared with 7 ICR control mice, 3 Ai140D;Rasgrf2-2A-dCre GFP-positive mice and 2 Ai140D-positive;Ragrf2-2A-dCre GFP-negative mice. Expression of GCaMP6f or GFP was induced by directly adding 10 μM TMP into growth medium of cultured neurons after dissection.

### Dissection and culturing of cortical neurons

Cortical neurons were dissected from newborn mice. Isolated tissues of cortex were digested with 0.25% trypsin for 15 min at 37°C. Then digestion was terminated by Dulbecco’s modified Eagle medium (DMEM) supplemented with 10% fetal bovine serum (FBS). The cell suspension was sieved through a filter and centrifuged at 1000 rpm for 5 min. The cell pellet was resuspended in DMEM supplemented with 10% FBS and were plated on poly-D-lysine-coated 35 mm No. 0 confocal dishes (In Vitro Scientific). After 4–6 hr, neurons were maintained in Neurobasal medium supplemented with 2% B27 and 1% glutaMAX-I (growth medium), and cultured in the incubator with temperature of 37°C and 5% CO_2_. Fresh growth medium was supplemented to neurons every 3–4 days to maintain the volume of 2 ml growth medium. All animals were obtained from the laboratory animal research center, Tsinghua University. Procedures involving animals has been approved by local institutional ethical committees (IACUC in Tsinghua University and Beihang University).

### Virus infection on cultured neurons

All viruses for infection of cultured neurons were provided by Hanbio Biotechnology, China. The neuron broad-spectrum promoter *Syn* and AAV2/DJ serotypes were selected for neuro-specific expression of GFP, GCaMP or GCaMP-X in cultured cortical neurons. 1 µl 1 × 10^12^ v.g./ml of the desired kinds of viruses were added to growth medium on DIV 0 unless otherwise noted. The same batches of cortical neurons were simultaneously observed for comparison. The expression of GCaMP and GCaMP-X was detectable on DIV 3, reached the peak on DIV 7 and sustained the high level up to 1 month. All experiments in vitro were repeated independently at least twice.

### Ca^2+^ imaging with GCaMP or GCaMP-X in cultured cortical neurons

Ca^2+^ imaging of neurons expressing GCaMP or GCaMP-X was acquired by confocal microscopy (A1RMP, Nikon, Japan; Dragonfly 200, Andor, England). 488 nm laser was used for excitation. 35 mm confocal dish containing cultured cortical neurons was set in the live-cell imaging culture chamber of the confocal microscope to maintain the environment of 37°C, 5% CO_2_ and ~95% humidity. Sampling rate of images was at 1–5 Hz and 3–5 view fields were selected from each dish. Fluorescence intensity (*F*) was subtracted from its background. *F*_0_ is the baseline fluorescence averaged from five data points at rest, and *ΔF* = *F-F*_*0*_. *ΔF*/*F*_*0*_ serves as the index for Ca^2+^ dynamics. Ca^2+^ waveforms were analyzed by the gadget of Quick Peaks in Origin software with the Three-Standard-Deviations Rule (values >3 SD). On and off rates were characterized by the time to rise up or decay down to 50% of the maximum (*ΔF*/*F*_*0*_), respectively. And the FWHM is defined as the duration of time between the (upward and downward) half-maximum timepoints. The mean of correlation coefficients based on Spearman Rank Correlation Coefficient in Origin software (Schaworonkow and Nikulin, 2019) was applied to quantify the degree of neuronal correlations based on all the traces of spontaneous Ca^2+^ signals per view (Sumi et al., 2020). FFT analyses were performed in Origin software. One-sided is selected for spectrum type and amplitude is define as below:

$$FFT\;Amplitude=2\sqrt{Re^{2}+Im^{2}}/n$$

Here, *Re* and *Im* are the real and imaginary parts of FFT results, and *n* is the size of the input signal.

Neurite tracings were depicted with Imaris 7.7.2 from the images under average intensity projection. Analyses on neuronal morphology and Ca^2+^ oscillations were performed with at least 30 neurons at each timepoint in each independent experiment. Fluorescence intensities of Ca^2+^ dynamics in neurons were color coded by Matlab (Mathworks) and Fiji.

### Transfection, confocal fluorescence imaging, and analysis of neurite morphology

2 μg of cDNA encoding jGCaMP7b, jGCaMP7b-X_C_, or jGCaMP7b-X_N_ and 1 μg cDNA encoding CFP (for labeling the soma area and neurites) were transiently transfected into DIV 5–7 cultured cortical neurons by Lipofectamine 2000 (Invitrogen) with a typical protocol according to the manual. The opti-MEM containing plasmids and Lipofectamine 2000 was added to the Neurobasal medium for transfection. After 2 hr, neurons were maintained in Neurobasal medium supplemented with 2% B27, 1% glutaMAX-I for at least 2 days before analyzing neurite morphology.

Fluorescence imaging of cultured cortical neurons was performed on ZEISS Laser Scanning Confocal Microscope (LSM710, Carl Zeiss) and ZEN 2009 software. N/C ratio of GCaMP or GCaMP-X was calculated by the ratio of fluorescence intensity (nuclear/cytosolic). Measurement of the total length and *Sholl* analysis for neurites were performed with Imaris 7.7.2 (Bitplane). Only non-overlapping neurons were selected for analysis and images of at least 24 neurons from two independent culture preparations were analyzed. Neurite tracings were depicted with Imaris 7.7.2 in CFP channel.

### Craniotomy and in vivo virus injection

Wildtype mice were used for virus injection and craniotomy under isoflurane anesthesia (5% for induction, 1–1.5% during surgery). AAV2/9-*Syn*-GCaMP6m-X_C_ (1.0 × 10^13^ v.g./ml, customized by BrainVTA, Wuhan, China) virus was tested in the primary somatosensory cortex (S1BF: AP −1.5, ML −3.0, DV 0.2/0.4, in mm), in comparison with AAV2/9-*Syn*-GCaMP6m (1.0 × 10^12^ v.g./ml, BrainVTA, Wuhan, China) virus as a control.

Craniotomy was done 3 weeks after virus injection. A piece of skull above S1BF was removed to expose a square imaging window (~3 × 3 mm, centered on S1BF) and the cortex was protected by a hand-cut glass window using #1 coverglass. Then the glass window was fixed using adhesive (Krazy glue, Elmer’s Products Inc) and dental cement. A head-post was also fixed to the posterior area of the mouse head using dental cement. 0.2 ml flunixin meglumine (0.25 mg/ml) was subcutaneously injected after surgery for 3 consecutive days.

### Preparations of brain slices and image analysis

Mice were anaesthetized by intraperitoneal injection of avertin solution (250–500 mg/kg). Then they were transcardially perfused with phosphate-buffered saline (PBS) followed by 4% PFA (paraformaldehyde) solution. Brains were immersed in 4% PFA solution overnight and were embedded in 2.5% agarose gel for slicing operation. Slices were obtained using the Lecia vibratome (LeciaVT1200S) with proper parameters including depth, speed and thickness of the brain section (50, 70, or 100 μm). Contrast of image was estimated by the equation below:

$$Contrast\;of\;image=1-\frac{2}{\left(F/F_{background}+1\right)}$$

### In vivo two-photon Ca^2+^ imaging

A two-photon random access mesoscope controlled with ScanImage 2017 (Vidrio Technologies) was used for in vivo Ca^2+^ imaging (Pologruto et al., 2003; Sofroniew et al., 2016). Images (512 × 512 pixels, 600 × 600, or 300 × 300 µm^2^) of L2/3 cells (150–250 µm under pia) in S1BF were collected at 7.4 Hz frame rate. Laser power (970 nm) was up to 60 mW out of objective. Calcium signal was extracted using CaImAn toolbox and data analysis was performed using Matlab (Giovannucci et al., 2019).

For functional test, imaging was carried out together with contralateral whisker stimulation using a 1.2-mm-diameter pole (~3 mm in amplitude, 10 vibrations in 0.5 s or 1 s for each trial). For each ROI, 20–40 trials were performed and calcium signaling was aligned to the onset of the whisker stimulation.

The fluorescence of each neuron was measured by averaging all pixels within the ROI (regions of interest) and corrected for neuropil contamination. The fluorescence signal was estimated by the equation below:

$$F_{cell}\left(t\right)=F_{measured}\left(t\right)-r*F_{neuropil}\left(t\right)$$

where *r* = 0.7 and *F*_neuropil_(*t*) was measured by averaging the fluorescence signal of all pixels within a 40 μm radium from the cell (Chen et al., 2013).

Signal-to-noise (SNR) was calculated as the ratio of *F*_max_/*F*_0_ to standard deviation of the filtered trace, 1 s period right before the whisker stimulate.

### Whole-cell electrophysiology

HEK293 cells (ATCC) were cultured in 60 mm dishes and checked by PCR with primers 5′-GGCGAATGGGTGAGTAACACG-3′ and 5′- CGGATAACGCTTGCGACCTATG-3′ to ensure free of mycoplasma contamination. Recombinant channels by α_1DL_, β_2a_ (M80545), and α_2_δ (NM012919.2) subunits (4 µg of cDNA for each) were transiently transfected according to established calcium phosphate protocol (Liu et al., 2017a; Liu et al., 2017b; Liu et al., 2010). To enhance expression, cDNA for simian virus 40T antigen (1 μg) was also cotransfected. Additional 2 μg of cDNA of jGCaMP7b or jGCaMP7b-X_C_ was added as required in cotransfections. Whole-cell recordings of transfected HEK293 cells were performed at room temperature (25°C) using an Axopatch 200B amplifier (Axon Instruments). The internal solutions contained (in mM): CsMeSO_3_, 135; CsCl, 5; MgCl_2_, 1; MgATP, 4; HEPES, 5; and EGTA, 5; at 290 mOsm adjusted with glucose and at pH 7.3 adjusted with CsOH. The extracellular solution contained (in mM): TEA-MeSO_3_, 135; HEPES, 10; CaCl_2_ or BaCl_2_, 10; 300 mOsm, adjusted with glucose and at pH 7.3 adjusted with TEAOH. Whole-cell currents were generated from a family of step depolarizations (−70 to +50 mV from a holding potential of −70 mV and step of 10 mV).

### Western blot

HEK293 cells or cortical neurons were washed by PBS three times, followed by being lysed in lysis buffer RIPA (radio immunoprecipitation assay) with protease inhibitor cocktail (Beyotime, P1006) for 20 min and centrifuged for 5 min at 14,000 × *g* at 4℃. Loading buffer was added to the supernatant. Then the sample were boiled for 7 min. Proteins were separated using 10% sodium dodecyl sulfate–polyacrylamide gel electrophoresis and transferred to a PVDF (polyvinylidene fluoride) membrane for 90 min. Then PVDF membrane was blocked in 5% non-fat dry milk and incubated with primary antibodies overnight at 4°C. Next, the PVDF membrane was washed three times with 1× TBST at room temperature with shaking, and incubated with secondary antibodies for 1–2 hr at room temperature then washed with 1× TBST for three times again. The membrane was coved with ECL chemiluminescent liquid (beyotime, P0018FM) before detection with an enhanced chemiluminescence system. Three or more independent replicates were performed for each experiment. Cytoplasmic proteins were extracted using nuclear extraction kit (Abcam, ab113474) following the instructions. Cytoplasmic proteins were collected by removing the nuclear proteins extracted via the kit.

### Coimmunoprecipitation assay

HEK293 cells were transfected by Lipofectamine and cultured for 2 days before cell lysates were prepared by lysis buffer RIPA (with protease inhibitor cocktail, Beyotime, P1006) and centrifugation at 14,000 × *g* for 5 min at 4℃. The supernatants were subjected to coimmunoprecipitation by using 20 µl anti-Flag or anti-Myc Magnetic Beads (Cat# B26102, B26301, bimake); and 5 mM EGTA overnight at 4°C. Beads were washed with PBST (1xPBS+0.5% Tween 20) for three times. Proteins were separated by sample loading buffer and boiled for 7 min. Then western blot was performed using the antibodies as indicated. Three or more independent replicates were performed for each experiment.

### Immunofluorescence staining

For immunostaining of cultured cortical neurons: Cortical neurons were fixed with PBS + 4% PFA for 15 min at room temperature, and washed three times by PBS. Fixed neurons were permeabilized in PBS + 0.3% Triton X-100 for 10 min, blocked in the 10% goat serum in PBS for 60 min, and then incubated in primary antibodies + 10% goat serum + PBS overnight in 4℃. Next day, cells were washed three times by PBS with gentle shaking, incubated in PBS + secondary antibodies for 60 min, and then washed three times by PBS with gentle shaking.

For immunostaining of brain slices: Sections after slicing (50 μm thickness) were immersed in 0.3% PBST solution for 15 min and the solution was renewed every 5 min. Then, sections were blocked in blocking solution (0.3% PBST solution with 3% bovine serum albumin) for 1 hr at room temperature and stained with primary antibodies at 4℃ for 36 hr. After washing three times by PBS, sections were incubated with secondary antibodies in turn for 60 min. Finally, sections were rinsed three times by PBS and prepared for visualization.

### Fluorescence Ca^2+^ imaging with Fluo-4 AM

Neurons were loaded with 2 μM Fluo-4 AM and 0.05% (wt/vol) Pluronic F127 in neurobasal medium at 37°C for 20 min in dark. Then neurons were gently washed twice with preheated 1× PBS. Neurons were incubated with neurobasal medium for 10 min in dark. Fluo-4 AM was excited at 488 nm, and emission signals were detected in 521 nm. Images were obtained using a ×20 objective with 512 × 512 pixels.

### Data analysis and statistics

Data were analyzed in Matlab, OriginPro, and GraphPad software. Standard error of the mean and two-tailed Student’s *t*-test or one-way analysis of variance followed by Bonferroni for post hoc tests were calculated when applicable. Criteria of significance: *p < 0.05; **p < 0.01; ***p < 0.001; and *n.s*. denotes ‘not significant’. All experiments were performed at least twice with appropriate sample sizes. Analyses of data were individually performed by at least three persons. Key experiments, such as the in vivo tests, were performed by one person and analyzed by other persons to avoid the potential bias.

## Data Availability

The plasmids of pEGFP-N1-jGCaMP7b-X_C_ (178361) and pEGFP-N1-jGCaMP7b-X_N_ (178362) are available on Addgene. Source data for WB and Co-IP are organized as four ZIP files. The data in details associated with the figures have been deposited to Dryad (https://doi.org/10.5061/dryad.zw3r22893). The following dataset was generated: GengJ TangY YuZ YangY GuoZ LiuX 2022Chronic Ca2+ imaging of cortical neurons with long-term expression of GCaMP-XDryad Digital Repository10.5061/dryad.zw3r22893PMC969969936196992

## Additional files

Transparent reporting form

## References

[bib1] Akerboom J, Chen TW, Wardill TJ, Tian L, Marvin JS, Mutlu S, Calderón NC, Esposti F, Borghuis BG, Sun XR, Gordus A, Orger MB, Portugues R, Engert F, Macklin JJ, Filosa A, Aggarwal A, Kerr RA, Takagi R, Kracun S, Shigetomi E, Khakh BS, Baier H, Lagnado L, Wang SSH, Bargmann CI, Kimmel BE, Jayaraman V, Svoboda K, Kim DS, Schreiter ER, Looger LL (2012). Optimization of a gcamp calcium indicator for neural activity imaging. The Journal of Neuroscience.

[bib2] Aoki R, Tsubota T, Goya Y, Benucci A (2017). An automated platform for high-throughput mouse behavior and physiology with voluntary head-fixation. Nature Communications.

[bib3] Aramuni G, Griesbeck O (2013). Chronic calcium imaging in neuronal development and disease. Experimental Neurology.

[bib4] Ben-Johny M, Yue DT (2014). Calmodulin regulation (calmodulation) of voltage-gated calcium channels. The Journal of General Physiology.

[bib5] Berridge MJ, Bootman MD, Roderick HL (2003). Calcium signalling: dynamics, homeostasis and remodelling. Nature Reviews. Molecular Cell Biology.

[bib6] Berridge MJ (2010). Calcium hypothesis of alzheimer’s disease. Pflugers Archiv.

[bib7] Chan CS, Guzman JN, Ilijic E, Mercer JN, Rick C, Tkatch T, Meredith GE, Surmeier DJ (2007). “Rejuvenation” protects neurons in mouse models of parkinson’s disease. Nature.

[bib8] Chen TW, Wardill TJ, Sun Y, Pulver SR, Renninger SL, Baohan A, Schreiter ER, Kerr RA, Orger MB, Jayaraman V, Looger LL, Svoboda K, Kim DS (2013). Ultrasensitive fluorescent proteins for imaging neuronal activity. Nature.

[bib9] Couto J, Musall S, Sun XR, Khanal A, Gluf S, Saxena S, Kinsella I, Abe T, Cunningham JP, Paninski L, Churchland AK (2021). Chronic, cortex-wide imaging of specific cell populations during behavior. Nature Protocols.

[bib10] Daigle TL, Madisen L, Hage TA, Valley MT, Knoblich U, Larsen RS, Takeno MM, Huang L, Gu H, Larsen R, Mills M, Bosma-Moody A, Siverts LA, Walker M, Graybuck LT, Yao Z, Fong O, Nguyen TN, Garren E, Lenz GH, Chavarha M, Pendergraft J, Harrington J, Hirokawa KE, Harris JA, Nicovich PR, McGraw MJ, Ollerenshaw DR, Smith KA, Baker CA, Ting JT, Sunkin SM, Lecoq J, Lin MZ, Boyden ES, Murphy GJ, da Costa NM, Waters J, Li L, Tasic B, Zeng H (2018). A suite of transgenic driver and reporter mouse lines with enhanced brain-cell-type targeting and functionality. Cell.

[bib11] Dana H, Sun Y, Mohar B, Hulse BK, Kerlin AM, Hasseman JP, Tsegaye G, Tsang A, Wong A, Patel R, Macklin JJ, Chen Y, Konnerth A, Jayaraman V, Looger LL, Schreiter ER, Svoboda K, Kim DS (2019). High-performance calcium sensors for imaging activity in neuronal populations and microcompartments. Nature Methods.

[bib12] Dolmetsch RE, Xu K, Lewis RS (1998). Calcium oscillations increase the efficiency and specificity of gene expression. Nature.

[bib13] Dutta S, Sengupta P (2016). Men and mice: relating their ages. Life Sciences.

[bib14] Estrada M, Uhlen P, Ehrlich BE (2006). Ca2+ oscillations induced by testosterone enhance neurite outgrowth. Journal of Cell Science.

[bib15] Fernández de Sevilla D, Garduño J, Galván E, Buño W (2006). Calcium-activated afterhyperpolarizations regulate synchronization and timing of epileptiform bursts in hippocampal CA3 pyramidal neurons. Journal of Neurophysiology.

[bib16] Garcia MI, Chen JJ, Boehning D (2017). Genetically encoded calcium indicators for studying long-term calcium dynamics during apoptosis. Cell Calcium.

[bib17] Gerendasy DD, Sutcliffe JG (1997). RC3/neurogranin, a postsynaptic calpacitin for setting the response threshold to calcium influxes. Molecular Neurobiology.

[bib18] Giovannucci A, Friedrich J, Gunn P, Kalfon J, Brown BL, Koay SA, Taxidis J, Najafi F, Gauthier JL, Zhou P, Khakh BS, Tank DW, Chklovskii DB, Pnevmatikakis EA (2019). CaImAn an open source tool for scalable calcium imaging data analysis. eLife.

[bib19] Gomez TM, Zheng JQ (2006). The molecular basis for calcium-dependent axon pathfinding. Nature Reviews. Neuroscience.

[bib20] Grødem S, Nymoen I, Vatne GH, Björnsdottir V, Lensjø KK, Fyhn M (2021). An updated suite of viral vectors for in vivo calcium imaging using local and retro-orbital injections. bioRxiv.

[bib21] Guzman JN, Sánchez-Padilla J, Chan CS, Surmeier DJ (2009). Robust pacemaking in substantia nigra dopaminergic neurons. The Journal of Neuroscience.

[bib22] Haiech J, Moreau M, Leclerc C, Kilhoffer MC (2019). Facts and conjectures on calmodulin and its cousin proteins, parvalbumin and troponin C. Biochimica et Biophysica Acta. Molecular Cell Research.

[bib23] Harr MW, Distelhorst CW (2010). Apoptosis and autophagy: decoding calcium signals that mediate life or death. Cold Spring Harbor Perspectives in Biology.

[bib24] Hong G, Lieber CM (2019). Novel electrode technologies for neural recordings. Nature Reviews. Neuroscience.

[bib25] Horigane S, Hamada S, Kamijo S, Yamada H, Yamasaki M, Watanabe M, Bito H, Ohtsuka T, Takemoto-Kimura S (2021). Development of an l-type ca2+ channel-dependent ca2+ transient during the radial migration of cortical excitatory neurons. Neuroscience Research.

[bib26] Huber D, Gutnisky DA, Peron S, O’Connor DH, Wiegert JS, Tian L, Oertner TG, Looger LL, Svoboda K (2012). Multiple dynamic representations in the motor cortex during sensorimotor learning. Nature.

[bib27] Isaev NK, Genrikhs EE, Voronkov DN, Kapkaeva MR, Stelmashook EV (2018). Streptozotocin toxicity in vitro depends on maturity of neurons. Toxicology and Applied Pharmacology.

[bib28] Ito D, Tamate H, Nagayama M, Uchida T, Kudoh SN, Gohara K (2010). Minimum neuron density for synchronized bursts in a rat cortical culture on multi-electrode arrays. Neuroscience.

[bib29] Kamijo S, Ishii Y, Horigane SI, Suzuki K, Ohkura M, Nakai J, Fujii H, Takemoto-Kimura S, Bito H (2018). A critical neurodevelopmental role for L-type voltage-gated calcium channels in neurite extension and radial migration. The Journal of Neuroscience.

[bib30] Khan TA, Revah O, Gordon A, Yoon S-J, Krawisz AK, Goold C, Sun Y, Kim CH, Tian Y, Li M-Y, Schaepe JM, Ikeda K, Amin ND, Sakai N, Yazawa M, Kushan L, Nishino S, Porteus MH, Rapoport JL, Bernstein JA, O’Hara R, Bearden CE, Hallmayer JF, Huguenard JR, Geschwind DH, Dolmetsch RE, Paşca SP (2020). Neuronal defects in a human cellular model of 22q11.2 deletion syndrome. Nature Medicine.

[bib31] Kirkby LA, Sack GS, Firl A, Feller MB (2013). A role for correlated spontaneous activity in the assembly of neural circuits. Neuron.

[bib32] Laviv T, Scholl B, Parra-Bueno P, Foote B, Zhang C, Yan L, Hayano Y, Chu J, Yasuda R (2020). In vivo imaging of the coupling between neuronal and CREB activity in the mouse brain. Neuron.

[bib33] Li W, Llopis J, Whitney M, Zlokarnik G, Tsien RY (1998). Cell-permeant caged insp3 ester shows that ca2+ spike frequency can optimize gene expression. Nature.

[bib34] Liebscher S, Keller GB, Goltstein PM, Bonhoeffer T, Hübener M (2016). Selective persistence of sensorimotor mismatch signals in visual cortex of behaving alzheimer’s disease mice. Current Biology.

[bib35] Liu X, Yang PS, Yang W, Yue DT (2010). Enzyme-inhibitor-like tuning of ca(2+) channel connectivity with calmodulin. Nature.

[bib36] Liu N, Liu Y, Yang Y, Liu X (2017a). Linker flexibility of IVS3-S4 loops modulates voltage-dependent activation of L-type ca^2+^ channels. Channels.

[bib37] Liu N, Yang Y, Ge L, Liu M, Colecraft HM, Liu X (2017b). Cooperative and acute inhibition by multiple C-terminal motifs of L-type ca^2+^ channels. eLife.

[bib38] Luhmann HJ, Sinning A, Yang JW, Reyes-Puerta V, Stüttgen MC, Kirischuk S, Kilb W (2016). Spontaneous neuronal activity in developing neocortical networks: from single cells to large-scale interactions. Frontiers in Neural Circuits.

[bib39] Madisen L, Garner AR, Shimaoka D, Chuong AS, Klapoetke NC, Li L, van der Bourg A, Niino Y, Egolf L, Monetti C, Gu H, Mills M, Cheng A, Tasic B, Nguyen TN, Sunkin SM, Benucci A, Nagy A, Miyawaki A, Helmchen F, Empson RM, Knöpfel T, Boyden ES, Reid RC, Carandini M, Zeng H (2015). Transgenic mice for intersectional targeting of neural sensors and effectors with high specificity and performance. Neuron.

[bib40] Mank M, Santos AF, Direnberger S, Mrsic-Flogel TD, Hofer SB, Stein V, Hendel T, Reiff DF, Levelt C, Borst A, Bonhoeffer T, Hübener M, Griesbeck O (2008). A genetically encoded calcium indicator for chronic in vivo two-photon imaging. Nature Methods.

[bib41] Mori N, Mook-Jung I (2015). Aging Mechanisms: Longevity, Metabolism, and Brain Aging.

[bib42] Moullan N, Mouchiroud L, Wang X, Ryu D, Williams EG, Mottis A, Jovaisaite V, Frochaux MV, Quiros PM, Deplancke B, Houtkooper RH, Auwerx J (2015). Tetracyclines disturb mitochondrial function across eukaryotic models: a call for caution in biomedical research. Cell Reports.

[bib43] Mukai Y, Shiina T, Jimbo Y (2003). Continuous monitoring of developmental activity changes in cultured cortical networks. Electrical Engineering in Japan.

[bib44] Murphy TH, Michelson NJ, Boyd JD, Fong T, Bolanos LA, Bierbrauer D, Siu T, Balbi M, Bolanos F, Vanni M, LeDue JM (2020). Automated task training and longitudinal monitoring of mouse mesoscale cortical circuits using home cages. eLife.

[bib45] Nakai J, Ohkura M, Imoto K (2001). A high signal-to-noise ca(2+) probe composed of A single green fluorescent protein. Nature Biotechnology.

[bib46] Nicotera P, Orrenius S (1998). The role of calcium in apoptosis. Cell Calcium.

[bib47] O’Banion CP, Yasuda R (2020). Fluorescent sensors for neuronal signaling. Current Opinion in Neurobiology.

[bib48] Obien MEJ, Frey U (2019). Large-scale, high-resolution microelectrode arrays for interrogation of neurons and networks. Advances in Neurobiology.

[bib49] Opitz T, De Lima AD, Voigt T (2002). Spontaneous development of synchronous oscillatory activity during maturation of cortical networks in vitro. Journal of Neurophysiology.

[bib50] Plumbly W, Brandon N, Deeb TZ, Hall J, Harwood AJ (2019). L-Type voltage-gated calcium channel regulation of in vitro human cortical neuronal networks. Scientific Reports.

[bib51] Pologruto TA, Sabatini BL, Svoboda K (2003). ScanImage: flexible software for operating laser scanning microscopes. Biomedical Engineering Online.

[bib52] Resendez SL, Jennings JH, Ung RL, Namboodiri VMK, Zhou ZC, Otis JM, Nomura H, McHenry JA, Kosyk O, Stuber GD (2016). Visualization of cortical, subcortical and deep brain neural circuit dynamics during naturalistic mammalian behavior with head-mounted microscopes and chronically implanted lenses. Nature Protocols.

[bib53] Rose T, Goltstein PM, Portugues R, Griesbeck O (2014). Putting a finishing touch on gecis. Frontiers in Molecular Neuroscience.

[bib54] Rosenberg SS, Spitzer NC (2011). Calcium signaling in neuronal development. Cold Spring Harbor Perspectives in Biology.

[bib55] Ruffinatti FA, Gilardino A, Lovisolo D, Ferraro M (2013). Spatial wavelet analysis of calcium oscillations in developing neurons. PLOS ONE.

[bib56] Sando R, Baumgaertel K, Pieraut S, Torabi-Rander N, Wandless TJ, Mayford M, Maximov A (2013). Inducible control of gene expression with destabilized CRE. Nature Methods.

[bib57] Schaworonkow N, Nikulin VV (2019). Spatial neuronal synchronization and the waveform of oscillations: implications for EEG and MEG. PLOS Computational Biology.

[bib58] Shafer TJ (2019). Application of microelectrode array approaches to neurotoxicity testing and screening. Advances in Neurobiology.

[bib59] Smith NA, Kress BT, Lu Y, Chandler-Militello D, Benraiss A, Nedergaard M (2018). Fluorescent ca^2+^ indicators directly inhibit the na,K-atpase and disrupt cellular functions. Science Signaling.

[bib60] Sofroniew NJ, Flickinger D, King J, Svoboda K (2016). A large field of view two-photon mesoscope with subcellular resolution for in vivo imaging. eLife.

[bib61] Spitzer NC (2006). Electrical activity in early neuronal development. Nature.

[bib62] Steinmetz NA, Buetfering C, Lecoq J, Lee CR, Peters AJ, Jacobs EAK, Coen P, Ollerenshaw DR, Valley MT, de Vries SEJ, Garrett M, Zhuang J, Groblewski PA, Manavi S, Miles J, White C, Lee E, Griffin F, Larkin JD, Roll K, Cross S, Nguyen TV, Larsen R, Pendergraft J, Daigle T, Tasic B, Thompson CL, Waters J, Olsen S, Margolis DJ, Zeng H, Hausser M, Carandini M, Harris KD (2017). Aberrant cortical activity in multiple gcamp6-expressing transgenic mouse lines. ENeuro.

[bib63] Sumi T, Yamamoto H, Hirano-Iwata A (2020). Suppression of hypersynchronous network activity in cultured cortical neurons using an ultrasoft silicone scaffold. Soft Matter.

[bib64] Sun JJ, Kilb W, Luhmann HJ (2010). Self-Organization of repetitive spike patterns in developing neuronal networks in vitro. The European Journal of Neuroscience.

[bib65] Takahashi N, Oertner TG, Hegemann P, Larkum ME (2016). Active cortical dendrites modulate perception. Science.

[bib66] Tallini YN, Ohkura M, Choi BR, Ji G, Imoto K, Doran R, Lee J, Plan P, Wilson J, Xin HB, Sanbe A, Gulick J, Mathai J, Robbins J, Salama G, Nakai J, Kotlikoff MI (2006). Imaging cellular signals in the heart in vivo: cardiac expression of the high-signal ca2+ indicator gcamp2. PNAS.

[bib67] Tian L, Hires SA, Mao T, Huber D, Chiappe ME, Chalasani SH, Petreanu L, Akerboom J, McKinney SA, Schreiter ER, Bargmann CI, Jayaraman V, Svoboda K, Looger LL (2009). Imaging neural activity in worms, flies and mice with improved gcamp calcium indicators. Nature Methods.

[bib68] Toth AB, Shum AK, Prakriya M (2016). Regulation of neurogenesis by calcium signaling. Cell Calcium.

[bib69] Uhlén P, Fritz N (2010). Biochemistry of calcium oscillations. Biochemical and Biophysical Research Communications.

[bib70] van Pelt J, Wolters PS, Corner MA, Rutten WLC, Ramakers GJA (2004). Long-Term characterization of firing dynamics of spontaneous bursts in cultured neural networks. IEEE Transactions on Bio-Medical Engineering.

[bib71] Vetter I, Carter D, Bassett J, Deuis JR, Tay B, Jami S, Robinson SD, Islam MS (2020). Calcium Signaling.

[bib72] Yang Y, Liu N, He Y, Liu Y, Ge L, Zou L, Song S, Xiong W, Liu X (2018). Improved calcium sensor gcamp-X overcomes the calcium channel perturbations induced by the calmodulin in gcamp. Nature Communications.

[bib73] Yang Y, Yu Z, Geng J, Liu M, Liu N, Li P, Hong W, Yue S, Jiang H, Ge H, Qian F, Xiong W, Wang P, Song S, Li X, Fan Y, Liu X (2022). Cytosolic peptides encoding ca_v_1 C-termini downregulate the calcium channel activity-neuritogenesis coupling. Communications Biology.

[bib74] Zariwala HA, Borghuis BG, Hoogland TM, Madisen L, Tian L, De Zeeuw CI, Zeng H, Looger LL, Svoboda K, Chen TW (2012). A CRE-dependent gcamp3 reporter mouse for neuronal imaging in vivo. The Journal of Neuroscience.

[bib75] Zhang Y, Rózsa M, Liang Y, Bushey D, Wei Z, Zheng J, Reep D, Broussard GJ, Tsang A, Tsegaye G, Narayan S, Obara CJ, Lim JX, Patel R, Zhang R, Ahrens MB, Turner GC, Wang SSH, Korff WL, Schreiter ER, Svoboda K, Hasseman JP, Kolb I, Looger LL (2021). Fast and Sensitive GCaMP Calcium Indicators for Imaging Neural Populations. bioRxiv.

