# Supplementary figures and images for: Chronic Ca2+ imaging of cortical neurons with long-term expression of GCaMP-X
Source: eLife. 2022 Oct 5;11:e76691. doi: 10.7554/eLife.76691 (PMC9699699; doi:10.7554/eLife.76691)

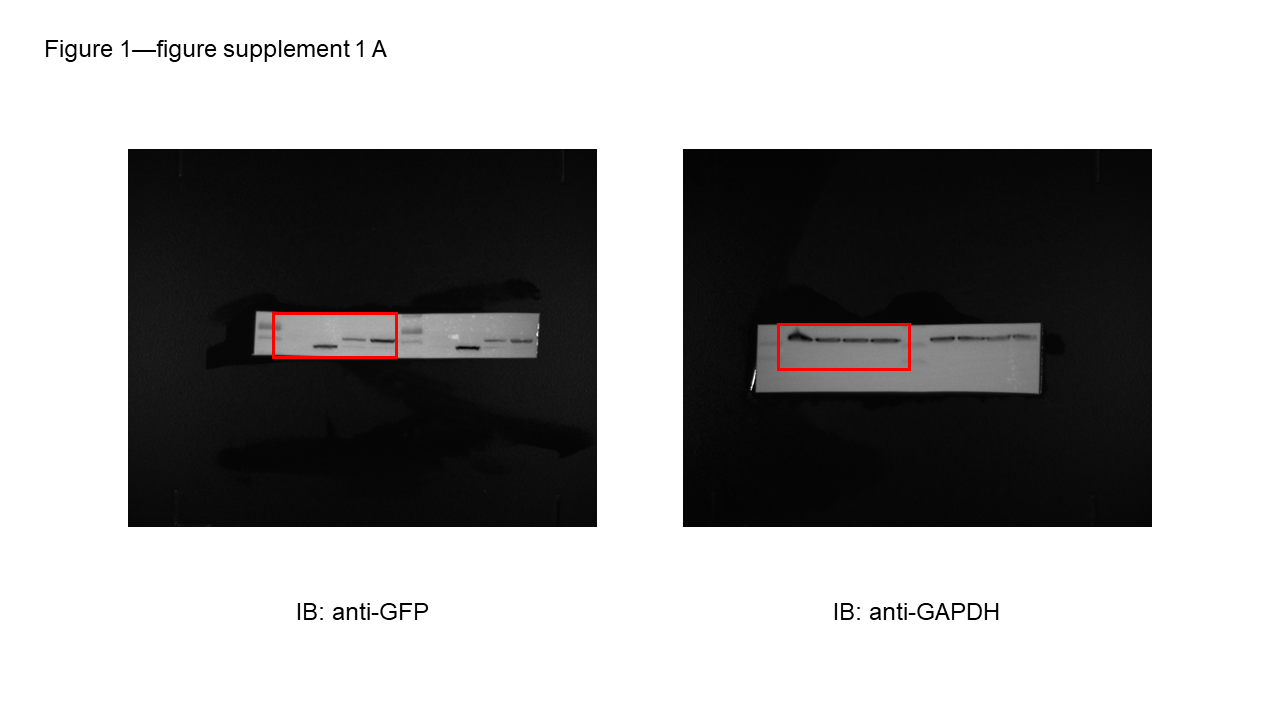

Supplement: Figure 1—figure supplement 1—source data 1. — Original uncropped western blotting gels with indication of the cropped areas. [file elife-76691-fig1-figsupp1-data1.zip › Figure 1-figure supplement 1-source data 1/F1S1A_annotated_blot.tif]

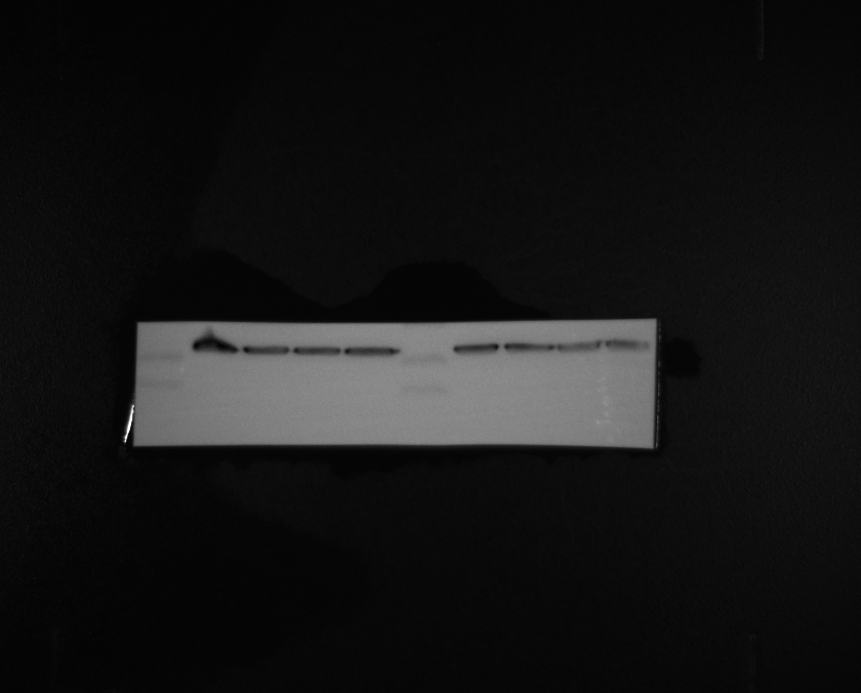

Supplement: Figure 1—figure supplement 1—source data 1. — Original uncropped western blotting gels with indication of the cropped areas. [file elife-76691-fig1-figsupp1-data1.zip › Figure 1-figure supplement 1-source data 1/F1S1A_GAPDH_raw_blot.tif]

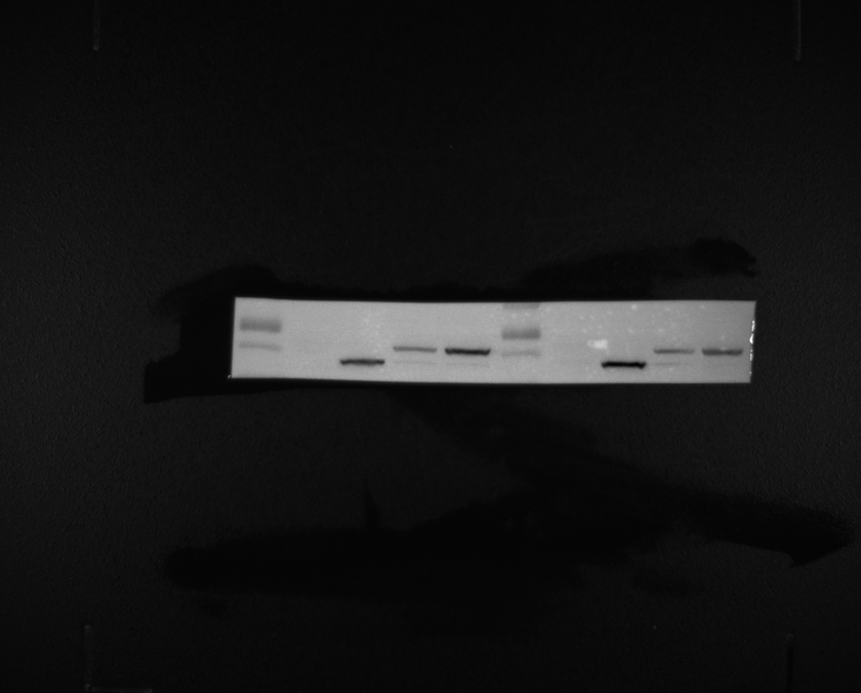

Supplement: Figure 1—figure supplement 1—source data 1. — Original uncropped western blotting gels with indication of the cropped areas. [file elife-76691-fig1-figsupp1-data1.zip › Figure 1-figure supplement 1-source data 1/F1S1A_GFP_raw_blot.tif]

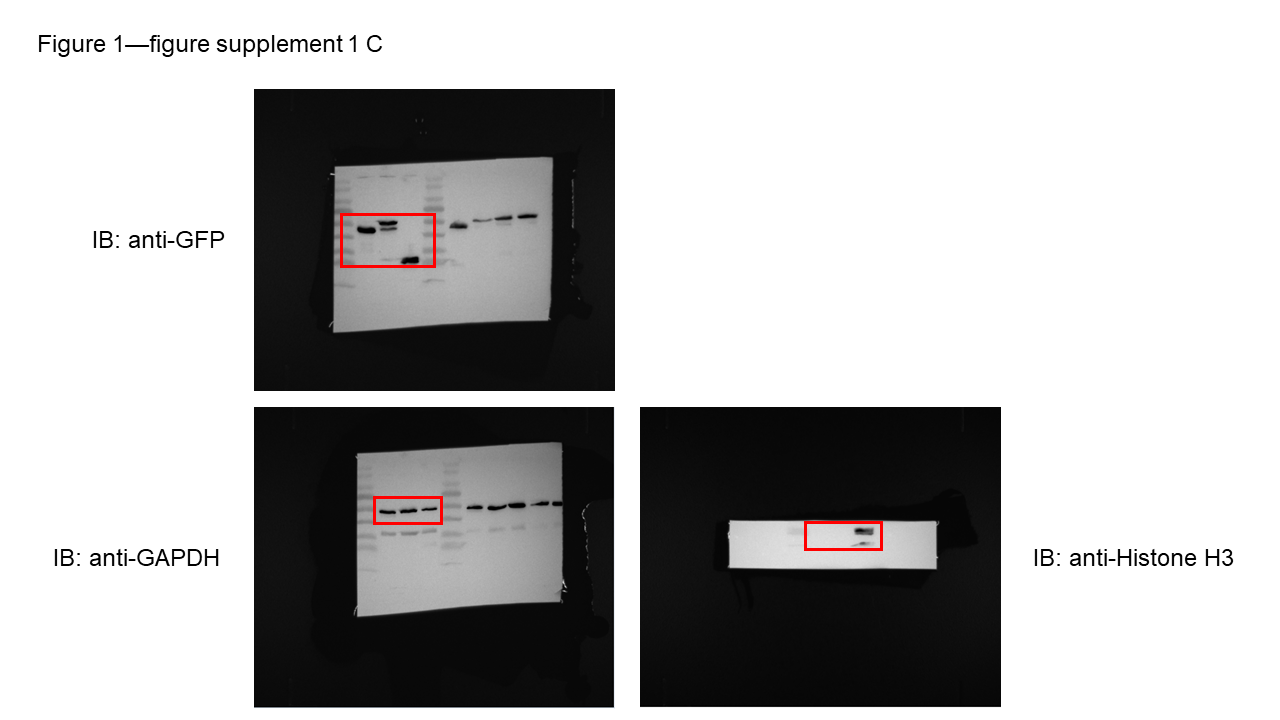

Supplement: Figure 1—figure supplement 1—source data 2. — Original uncropped western blotting gels with indication of the cropped areas. [file elife-76691-fig1-figsupp1-data2.zip › Figure 1-figure supplement 1-source data 2/F1S1C_annotated_blot.tif]

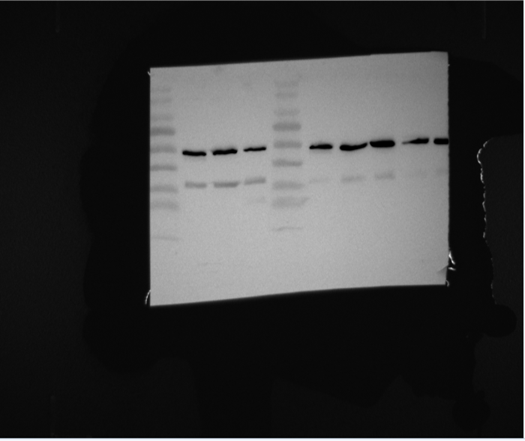

Supplement: Figure 1—figure supplement 1—source data 2. — Original uncropped western blotting gels with indication of the cropped areas. [file elife-76691-fig1-figsupp1-data2.zip › Figure 1-figure supplement 1-source data 2/F1S1C_GAPDH_raw_blot.tif]

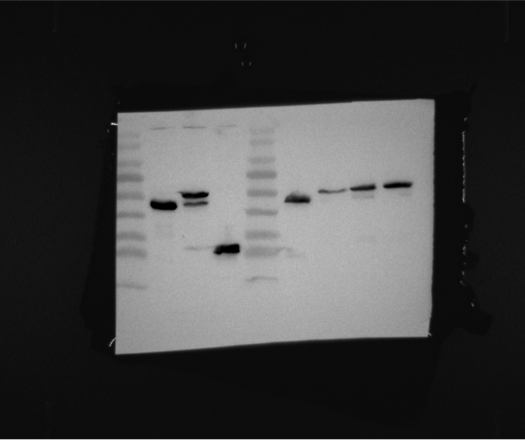

Supplement: Figure 1—figure supplement 1—source data 2. — Original uncropped western blotting gels with indication of the cropped areas. [file elife-76691-fig1-figsupp1-data2.zip › Figure 1-figure supplement 1-source data 2/F1S1C_GFP_raw_blot.tif]

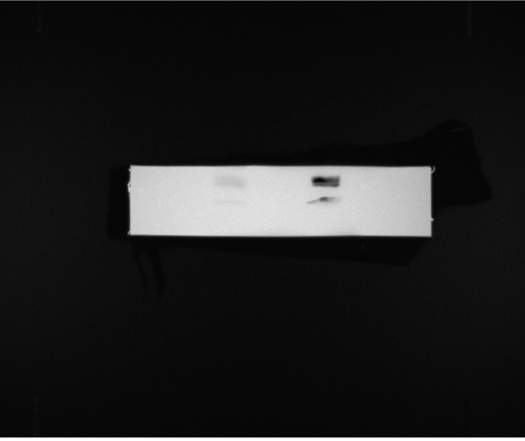

Supplement: Figure 1—figure supplement 1—source data 2. — Original uncropped western blotting gels with indication of the cropped areas. [file elife-76691-fig1-figsupp1-data2.zip › Figure 1-figure supplement 1-source data 2/F1S1C_H3_raw_blot.tif]

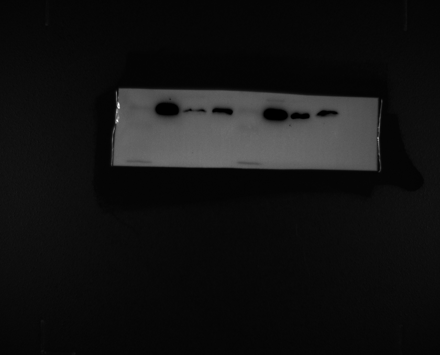

Supplement: Figure 1—figure supplement 3—source data 1. — Original uncropped western blotting gels with indication of the cropped areas. [file elife-76691-fig1-figsupp3-data1.zip › Figure 1-figure supplement 3-source data 1/F1S3A_Flag_raw_blot.tif]

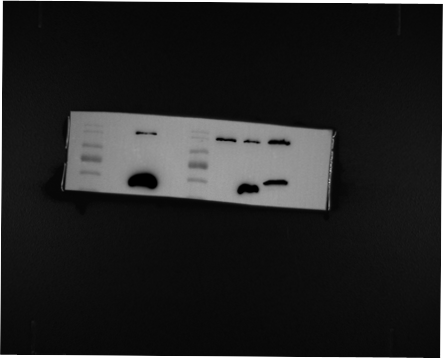

Supplement: Figure 1—figure supplement 3—source data 1. — Original uncropped western blotting gels with indication of the cropped areas. [file elife-76691-fig1-figsupp3-data1.zip › Figure 1-figure supplement 3-source data 1/F1S3A_His_raw_blot.tif]

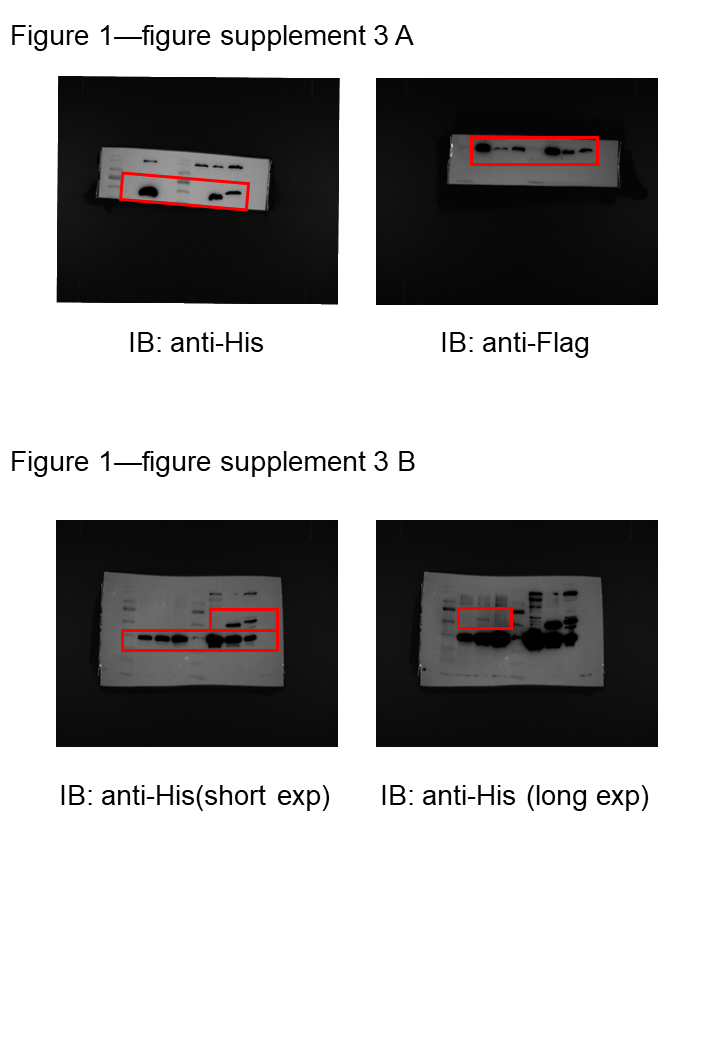

Supplement: Figure 1—figure supplement 3—source data 1. — Original uncropped western blotting gels with indication of the cropped areas. [file elife-76691-fig1-figsupp3-data1.zip › Figure 1-figure supplement 3-source data 1/F1S3AB_annotated_blot.tif]

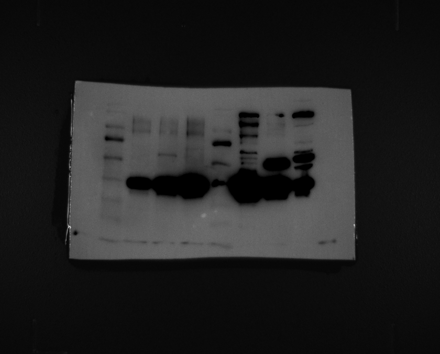

Supplement: Figure 1—figure supplement 3—source data 1. — Original uncropped western blotting gels with indication of the cropped areas. [file elife-76691-fig1-figsupp3-data1.zip › Figure 1-figure supplement 3-source data 1/F1S3B_His_long exp_raw_blot.tif]

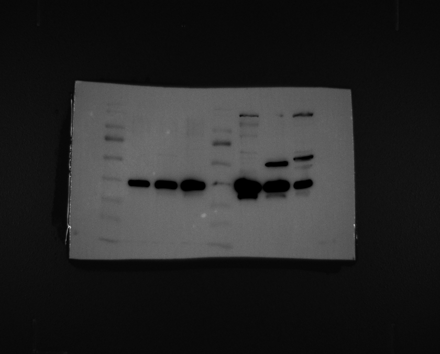

Supplement: Figure 1—figure supplement 3—source data 1. — Original uncropped western blotting gels with indication of the cropped areas. [file elife-76691-fig1-figsupp3-data1.zip › Figure 1-figure supplement 3-source data 1/F1S3B_His_short exp_raw_blot.tif]

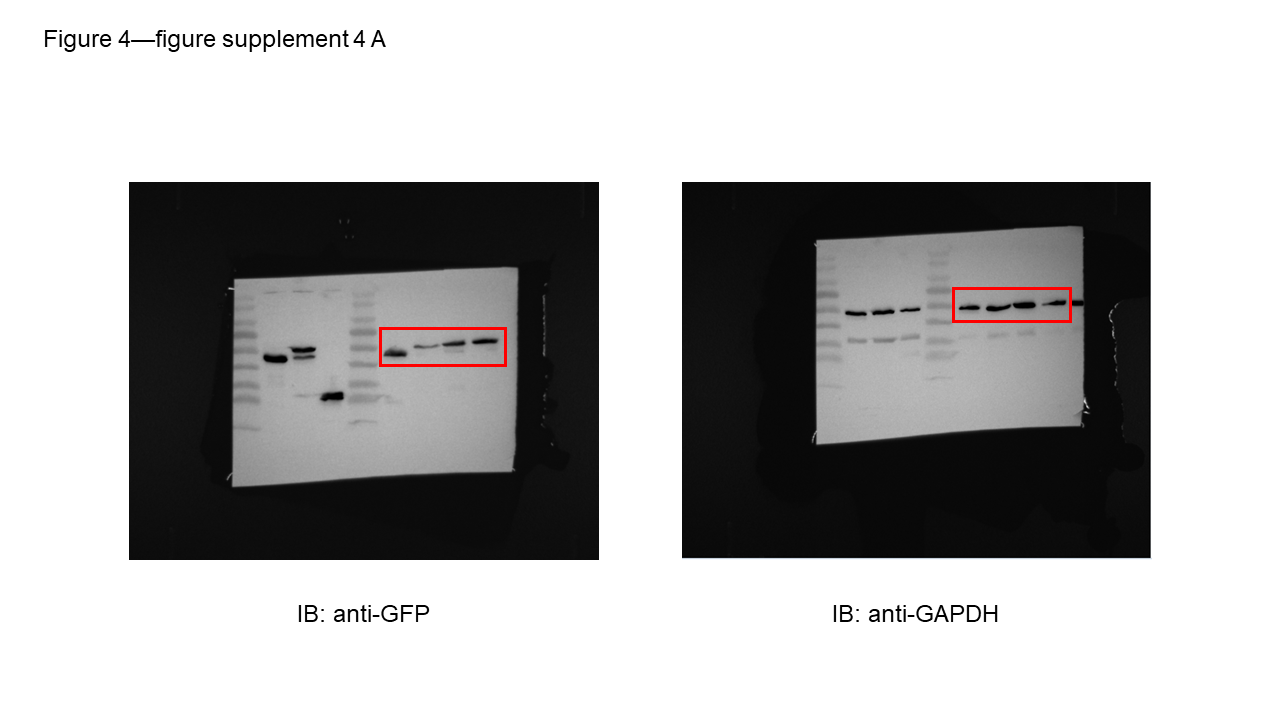

Supplement: Figure 4—figure supplement 4—source data 1. — Original uncropped western blotting gels with indication of the cropped areas. [file elife-76691-fig4-figsupp4-data1.zip › Figure 4-figure supplement 4-source data 1/F4S4A_annotated_blot.tif]

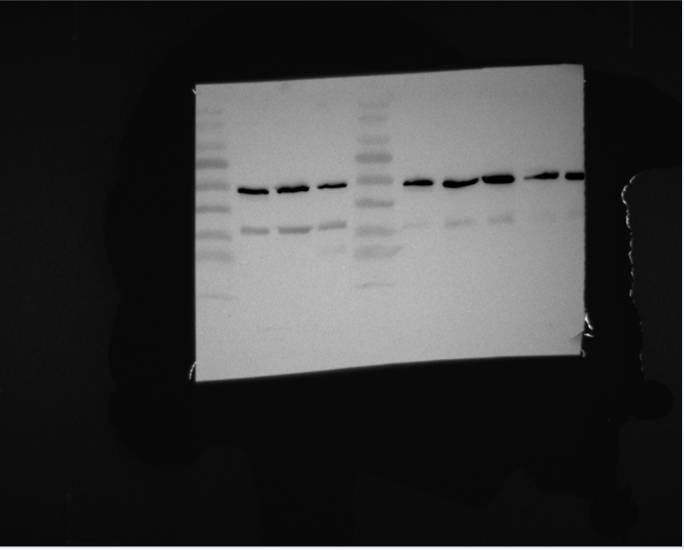

Supplement: Figure 4—figure supplement 4—source data 1. — Original uncropped western blotting gels with indication of the cropped areas. [file elife-76691-fig4-figsupp4-data1.zip › Figure 4-figure supplement 4-source data 1/F4S4A_GAPDH_raw-blot.tif]

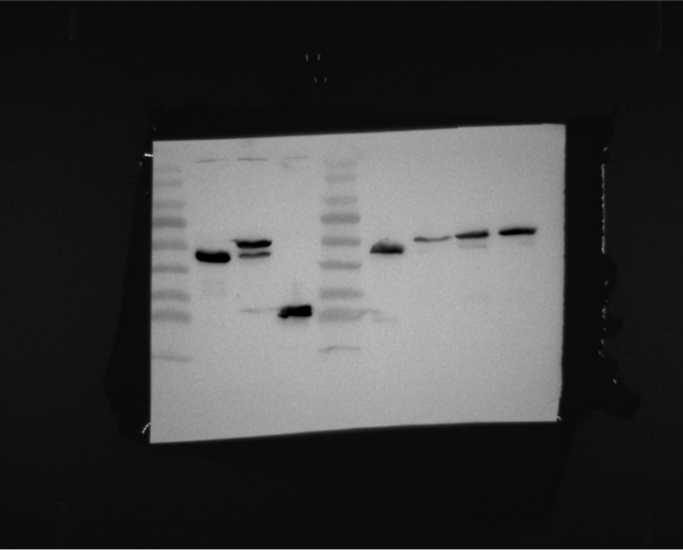

Supplement: Figure 4—figure supplement 4—source data 1. — Original uncropped western blotting gels with indication of the cropped areas. [file elife-76691-fig4-figsupp4-data1.zip › Figure 4-figure supplement 4-source data 1/F4S4A_GFP_raw-blot.tif]
